# Disruption and change in positivity of respiratory pathogen epidemiology in children during and after the COVID-19 pandemic, 2018–2023: a large-scale period-comparison study in Guangzhou, southern China

**DOI:** 10.3389/fmicb.2026.1992791

**Published:** 2026-10-20

**Authors:** Huan Li, Ru Bai, Zishan Tang, Ping Li, Lingling Tang, Xianhua Zheng, Chunming Gu, Cunwei Ji, Yasha Luo, Mingyong Luo

**Affiliations:** 1 Department of Clinical Laboratory, Guangdong Women and Children Hospital, Guangzhou, China; 2 Graduate School, Guangzhou Medical University, Guangzhou, China

**Keywords:** age stratification, COVID-19, hypothesized immunity debt, influenza, *Mycoplasma pneumoniae*, pediatrics, respiratory pathogens, RSV

## Abstract

**Background:**

Non-pharmaceutical interventions (NPIs) during the COVID-19 pandemic disrupted respiratory pathogen transmission, but post-pandemic change in positivity patterns across pathogens, age groups, and co-infections remain incompletely characterized in children.

**Methods:**

We conducted a retrospective period-comparison study at two Guangzhou hospitals, encompassing 230,333 patient-period records (2018–2023). Using logistic regression, we compared adjusted odds ratios (aOR) for 16 pathogens across three pandemic periods (Pandemic-I: strict NPIs; Pandemic-II: relaxed NPIs; Post-pandemic: full reopening) versus pre-pandemic baseline, with age-period interactions tested by likelihood-ratio.

**Results:**

Most respiratory viruses and *M. pneumoniae* were suppressed in Pandemic-I, with notable exceptions such as rhinovirus, CMV, and bocavirus (influenza A aOR = 0.23; B aOR = 0.08). Post-pandemic changes in test positivity diverged: influenza A showed increased positivity across all ages (aOR = 1.23–1.74); RSV showed increased positivity in children <5 years (aOR = 1.50–1.86); *M. pneumoniae* showed a marked increase in positivity in school-aged children (5–9 years: aOR = 3.32); influenza B remained suppressed in young children (aOR = 0.53–0.58) but showed increased positivity in adolescents (aOR = 2.88). Age-period interactions were significant for all four (*p* < 0.001). Bacterial changes were modest and should be interpreted cautiously because culture positivity is influenced by specimen type, hospitalization, antibiotic exposure, and culture indications. Co-infections shifted from bacterial–bacterial dominance in Pandemic-I (49.0%) to bacterial–influenza co-infections post-pandemic (22.9%).

**Conclusion:**

The pandemic reshaped pediatric respiratory pathogen epidemiology in pathogen- and age-specific ways, and may warrant consideration of age-stratified surveillance and tailored prevention.

## 1 Introduction

The COVID-19 pandemic, driven by SARS-CoV-2, has precipitated an unprecedented global health crisis since its emergence in late 2019. In response, countries worldwide implemented stringent non-pharmaceutical interventions (NPIs), including mask mandates, social distancing, school closures, and travel restrictions, which effectively curtailed the transmission of SARS-CoV-2 (Flaxman et al., 2020; Lai et al., 2020). However, these measures also exerted profound and unintended effects on the epidemiology of other respiratory pathogens (Chow et al., 2023; Olsen et al., 2021).

Numerous studies have documented sharp declines in influenza, respiratory syncytial virus (RSV), and other endemic respiratory viruses during the early pandemic period (Britton et al., 2020; Huang et al., 2021; Kuitunen et al., 2020; Li Z. J. et al., 2022). Yet, as NPIs were relaxed and populations resumed normal activities, many of these pathogens rebounded a phenomenon increasingly hypothesized to reflect a “potential immunity gap”, although serological data were not available in the present study to test this mechanism. Resulting from reduced pathogen exposure during the pandemic (Cohen et al., 2021; Messacar et al., 2022). Subsequent studies have further characterized the resurgent patterns of specific pathogens, particularly RSV and influenza, across diverse geographic settings (Li Y. et al., 2022).

Children represent a particularly vulnerable population, given their high susceptibility to respiratory infections and their central role in community transmission (Viner et al., 2021). However, most existing studies have focused on single pathogens or short observation windows, and comprehensive analyses encompassing viruses, atypical bacteria, and common bacteria across the full pandemic-to-post-pandemic continuum remain scarce (Bardsley et al., 2023). Moreover, whether the effect of NPIs and subsequent change in positivity is uniform across pediatric age strata, and whether the interaction between age and pandemic period exists, has not been systematically examined for a broad panel of respiratory pathogens.

To address these gaps, we conducted a large-scale retrospective period-comparison study spanning 6 years (2018–2023) at two tertiary pediatric hospitals in Guangzhou, China. We aimed to: (1) characterize the dynamic changes in positivity rates and adjusted risks for 16 respiratory pathogens across three distinct pandemic periods relative to the pre-pandemic baseline; (2) examine whether these effects are modified by age group; and (3) describe the evolving patterns of co-infections across periods and age strata. Our findings may inform age-specific surveillance strategies and clinical preparedness for future epidemic works.

## 2 Materials and methods

### 2.1 Study design and setting

This retrospective study was conducted at two tertiary teaching hospitals located in Panyu district and Yuexiu district in Guangzhou, Guangdong Province, China. Both hospitals serve as major referral centers for pediatric respiratory diseases in the Pearl River Delta region.

### 2.2 Study period definition

To capture the dynamic changes in respiratory pathogen epidemiology across the full spectrum of the COVID-19 pandemic, we defined a 6-year observation window from January 1, 2018, to December 31, 2023. This interval was partitioned into four consecutive periods according to the evolving local COVID-19 containment strategies (Flaxman et al., 2020). The pre-pandemic baseline period spanned from January 2018 to January 2020, during which no NPIs were in place and no community transmission of SARS-CoV-2 was documented. The subsequent pandemic period was further stratified into four distinct phases: Pre-pandemic (Jan 2018-Jan 2020); Pandemic-I (February 2020–June 2021), characterized by stringent NPIs including lockdowns, school closures, universal masking, and travel bans, with negligible local SARS-CoV-2 transmission; Pandemic-II (July 2021–November 2022), marked by relaxed NPIs with targeted restrictions and gradual reopening, during which intermittent community outbreaks of the Omicron variant occurred; and the Post-pandemic period (December 2022–December 2023), defined as full reopening following the national policy shift in December 2022. Locally, Guangdong Province launched a Level 1 public health emergency response on January 23, 2020; Guangzhou subsequently implemented the response locally (People’s Daily Online, January 23, 2020)^1^, followed by a Delta variant outbreak in May-June 2021, and a full reopening in November-December 2022.

### 2.3 Study population

We included all pediatric patients (aged 0–17 years) who visited either hospital and underwent testing for any of the targeted respiratory pathogens during the study period. A “patient-period” was defined as one patient’s testing record within a single pandemic period; patients with multiple visits across different periods were counted separately for each period. Within a given patient-period, multiple tests were combined such that any positive result classified the patient-period as positive for viral pathogens. For bacterial culture, when multiple specimen types were available, lower-respiratory specimens were prioritized because they provide higher culture yield; if only upper-respiratory specimens were available, the upper-respiratory result was used. The study flow, including the number of records excluded at each step, is shown in Supplementary Figure 1. The net reduction from 541,671 test records to 230,333 patient-periods reflects both exclusions and aggregation of multiple tests from the same child within a period. The total number of unique children (197,630) is smaller than the total number of patient-period records (230,333), reflecting repeated contribution of some children across periods.

Exclusion criteria: (1) patients with incomplete demographic or clinical data; (2) duplicate records; (3) tests performed for surveillance rather than clinical indications.

### 2.4 Pathogen testing and data collection

Respiratory specimens (nasopharyngeal swabs, throat swabs, sputum, or bronchoalveolar lavage fluid) were tested for a broad range of pathogens. Influenza A and B were identified by rapid antigen testing targeting viral nucleoprotein (Wondfo, Guangzhou, China). RSV, bocavirus, CMV, EBV, rhinovirus, and enterovirus were detected by multiplex real-time fluorescence PCR (DaAn Gene, Guangzhou, China), targeting conserved regions of the respective viral genomes; adenovirus was detected by real-time fluorescence PCR targeting the hexon and penton genes (Sansure Biotech, Changsha, China). *M. pneumoniae* was detected by isothermal amplification (Ustar, Hangzhou, China) throughout the study period. No serological testing for *M. pneumoniae* was performed at any point during the study; all *M. pneumoniae* results were based on nucleic acid amplification from respiratory specimens, and paired serology or antibody titers were not collected. *M. tuberculosis* was detected by real-time fluorescence PCR (DaAn Gene, Guangzhou, China),smear and culture were not used for the study endpoint. Common bacteria were identified by routine culture. The assays and platforms used for each pathogen remained unchanged across the four study periods; however, the number of children tested changed substantially over time, especially for RSV and *M. pneumoniae*. Clinical data, including age, sex, care setting, hospital campus, specimen type, length of stay, and hospitalization costs, were obtained from electronic medical records.

### 2.5 Statistical analysis

All statistical analyses were performed using R version 4.2.2 (R Foundation for Statistical Computing, Vienna, Austria). A two-sided *p* < 0.05 was considered statistically significant.

Categorical variables were presented as frequencies and percentages, with comparisons across periods using chi-square tests. Continuous variables were summarized as median with interquartile range (IQR) and compared using Kruskal–Wallis tests.

The primary endpoint of this study is positivity among tested children, not community incidence. Expansion of testing may introduce many mildly symptomatic or asymptomatic children with low pathogen load, thereby diluting crude positivity rates. Conversely, targeted testing in inpatients may enrich for true cases and increase positivity. Adjusted analyses and sensitivity analyses restricted to more consistently tested subgroups were used to mitigate this bias. For each pathogen, we calculated crude odds ratios (OR) for each pandemic period versus the pre-pandemic reference using univariable logistic regression. Estimates were not reported (marked “NE”) if the number of positive events was <5 or the 95% confidence interval (CI) width exceeded 10-fold.

To account for potential within-patient correlation, we repeated the main analyses using cluster-robust standard errors by child. Results were virtually unchanged compared with the primary models, consistent with the low proportion of children contributing to more than one period.

Multivariable logistic regression was performed for each pathogen, adjusting for age (as a categorical variable: 0–28 days, 29 days–23 months, 2–4 years, 5–9 years, 10–17 years), sex, care setting (inpatient/outpatient), and hospital campus. For bacterial culture analyses, the primary model excluded specimen type because it is a near-deterministic mediator of culture yield that shifted markedly across periods (upper-respiratory specimens increased from 91.9% pre-pandemic to 99.2% post-pandemic), and lower-respiratory specimens yielded substantially higher culture positivity (e.g., *P. aeruginosa* 13.6×, *E. coli* 8.7×; Supplementary Table 16). A sensitivity model including specimen type is provided in Supplementary Table 12; however, because specimen type lies on the causal pathway between pandemic period and culture positivity, adjusting for it may introduce over-adjustment bias, and neither estimate should be regarded as definitively preferable. Bacterial culture positivity is also influenced by inpatient proportion, prior antibiotic exposure, and culture indications, all of which changed over the study period. These factors may confound the interpretation of bacterial trends and should be considered when comparing culture positivity across periods. For viral analyses, specimen type was excluded to avoid over-adjustment.

The four most-disrupted pathogens (RSV, influenza A, influenza B, and *M. pneumoniae*) were selected *post hoc* as primary endpoints. We performed separate multivariable logistic regression models within each age stratum, adjusting for sex, care setting, and hospital campus. Age–period interactions were evaluated using robust Wald tests based on cluster-robust variance estimates.

Baseline characteristics were also described separately for patients tested by each major diagnostic modality (bacterial culture, viral PCR, and influenza antigen) to account for shifting testing populations over time.

### 2.6 Ethics approval

This study was approved by the Institutional Review Board of Guangdong Women and Children Hospital (Approval No. 202201044). Due to hospital data privacy regulations, the raw patient data cannot be made publicly available. Researchers may request access to the de-identified data from the corresponding author, subject to institutional review board approval. The requirement for informed consent was waived due to the retrospective nature of the study, and all data were anonymized prior to analysis. A data dictionary is provided as Supplementary Table 17.

## 3 Results

### 3.1 Baseline characteristics

A total of 230,333 patient-period records from 197,630 unique children were included in the analysis. Because some children contributed to more than one period, cluster-robust standard errors by child were used in sensitivity analyses; the results were virtually identical to the primary models. The demographic and clinical characteristics of the study population varied significantly across the four periods (Table 1). The age distribution shifted substantially over time. Specifically, young children aged 29 days–23 months constituted the largest group in the pre-pandemic period (36.7%), whereas school-aged children (5–9 years) increased from 21.8% pre-pandemic to 34.9% post-pandemic, and adolescents (10–17 years) rose from 5.3% to 12.6% (*p* < 0.001). Male sex was consistently predominant across all periods (56.4%–58.0%). The proportion of inpatients increased during Pandemic-I (22.3%) and Pandemic-II (27.3%) compared with baseline (16.2%), then returned to 15.1% post-pandemic (*p* < 0.001). Upper respiratory tract specimens comprised 91.9% of all samples pre-pandemic and increased progressively to 99.2% post-pandemic (*p* < 0.001). Among inpatients only, the median length of hospital stay decreased from 8 days pre-pandemic to 7 days post-pandemic (*p* < 0.001), while hospitalization costs remained relatively stable across periods (median range: ¥7,078–8,414). Baseline characteristics of patients stratified by testing modality (bacterial culture, viral PCR, and influenza antigen test) are presented in Supplementary Tables 1–3. Monthly test volumes are shown in Figure 1; denominators changed substantially for RSV and *M. pneumoniae*, reflecting the shift from selective to routine testing over time. These changes in testing practice should be considered when interpreting crude positivity rates.

**TABLE 1:** Baseline characteristics by pandemic period (all patients).

Variable	Level	Pre-pandemic	Pandemic-I	Pandemic-II	Post-pandemic	Overall	*P*
*N* (patient-periods)		88,710	15,443	56,393	69,787	230,333	
Unique children		88,710	15,443	56,393	69,787	197,630	
Hospital campus	Panyu	54,842 (61.8)	9,204 (59.6)	37,198 (66.0)	44,525 (63.8)	145,769 (63.3)	<0.001
	YX	33,868 (38.2)	6,239 (40.4)	19,195 (34.0)	25,262 (36.2)	84,564 (36.7)	
Care setting	Inpatient	14,338 (16.2)	3,444 (22.3)	15,400 (27.3)	10,507 (15.1)	43,689 (19.0)	<0.001
	Outpatient	74,372 (83.8)	11,999 (77.7)	40,993 (72.7)	59,280 (84.9)	186,644 (81.0)	
Sex	Female	37,690 (42.5)	6,495 (42.1)	23,677 (42.0)	30,448 (43.6)	98,310 (42.7)	<0.001
	Male	51,020 (57.5)	8,948 (57.9)	32,716 (58.0)	39,339 (56.4)	132,023 (57.3)	
Specimen	Upper resp.	81,538 (91.9)	13,657 (88.4)	52,434 (93.0)	69,238 (99.2)	216,867 (94.2)	<0.001
	Lower resp.	7,172 (8.1)	1,786 (11.6)	3,959 (7.0)	549 (0.8)	13,466 (5.8)	
Age group	0–28 d	2,551 (2.9)	754 (4.9)	5,762 (10.2)	3,689 (5.3)	12,756 (5.5)	<0.001
	29 d–23 mo	32,532 (36.7)	6,763 (43.8)	13,040 (23.1)	12,293 (17.6)	64,628 (28.1)	
	2–4 y	29,584 (33.3)	4,963 (32.1)	19,046 (33.8)	20,658 (29.6)	74,251 (32.2)	
	5–9 y	19,373 (21.8)	2,215 (14.3)	14,466 (25.7)	24,347 (34.9)	60,401 (26.2)	
	10–17 y	4,670 (5.3)	748 (4.8)	4,079 (7.2)	8,800 (12.6)	18,297 (7.9)	
Year*	2018	30,297 (34.2)	0 (0.0)	0 (0.0)	0 (0.0)	30,297 (13.2)	<0.001
	2019	53,447 (60.2)	0 (0.0)	0 (0.0)	0 (0.0)	53,447 (23.2)	
	2020	4,966 (5.6)	14,591 (94.5)	0 (0.0)	0 (0.0)	19,557 (8.5)	
	2021	0 (0.0)	852 (5.5)	15,977 (28.3)	0 (0.0)	16,829 (7.3)	
	2022	0 (0.0)	0 (0.0)	40,416 (71.7)	717 (1.0)	41,133 (17.9)	
	2023	0 (0.0)	0 (0.0)	0 (0.0)	69,070 (99.0)	69,070 (30.0)	
Length of stay, d (inpatients)	Median [IQR]	8 [6–11]	7 [6–10]	6 [5–9]	7 [5–9]	7 [5–10]	<0.001
	Inpatients with data, n	14,338	3,444	15,400	10,450	43,632	
Hospital cost, ¥ (inpatients)	Median [IQR]	8414 [5143–13650]	7078 [4172–12137]	7327 [4456–11333]	7546 [4716–10055]	7698 [4700–11635]	<0.001
	Inpatients with data, *n*	14,337	3,444	15,400	10,507	43,688	

Values are *n* (%) unless stated. *P* from chi-square (categorical) or Kruskal–Wallis (continuous). Analysis unit: patient-period (one record per patient per period); unique children are also shown. Periods were defined as: pre-pandemic, January 2018–January 2020; Pandemic-I, February 2020–June 2021; Pandemic-II, July 2021–November 2022; post-pandemic, December 2022–December 2023. Unique children were counted within each period; because a child may contribute to more than one period, the sum across periods exceeds the overall number of unique children (197,630). Length of stay and hospital cost are restricted to inpatients with observed data (outpatients have no admission).

*Year rows show calendar-year totals. Because periods were defined by month, the 2020 row includes January 2020 for the pre-pandemic period and February-December 2020 for Pandemic-I; the 2021 row includes January-June 2021 for Pandemic-I and July-December 2021 for Pandemic-II; the 2022 row includes January-November 2022 for Pandemic-II and December 2022 for the post-pandemic period.

**FIGURE 1:**
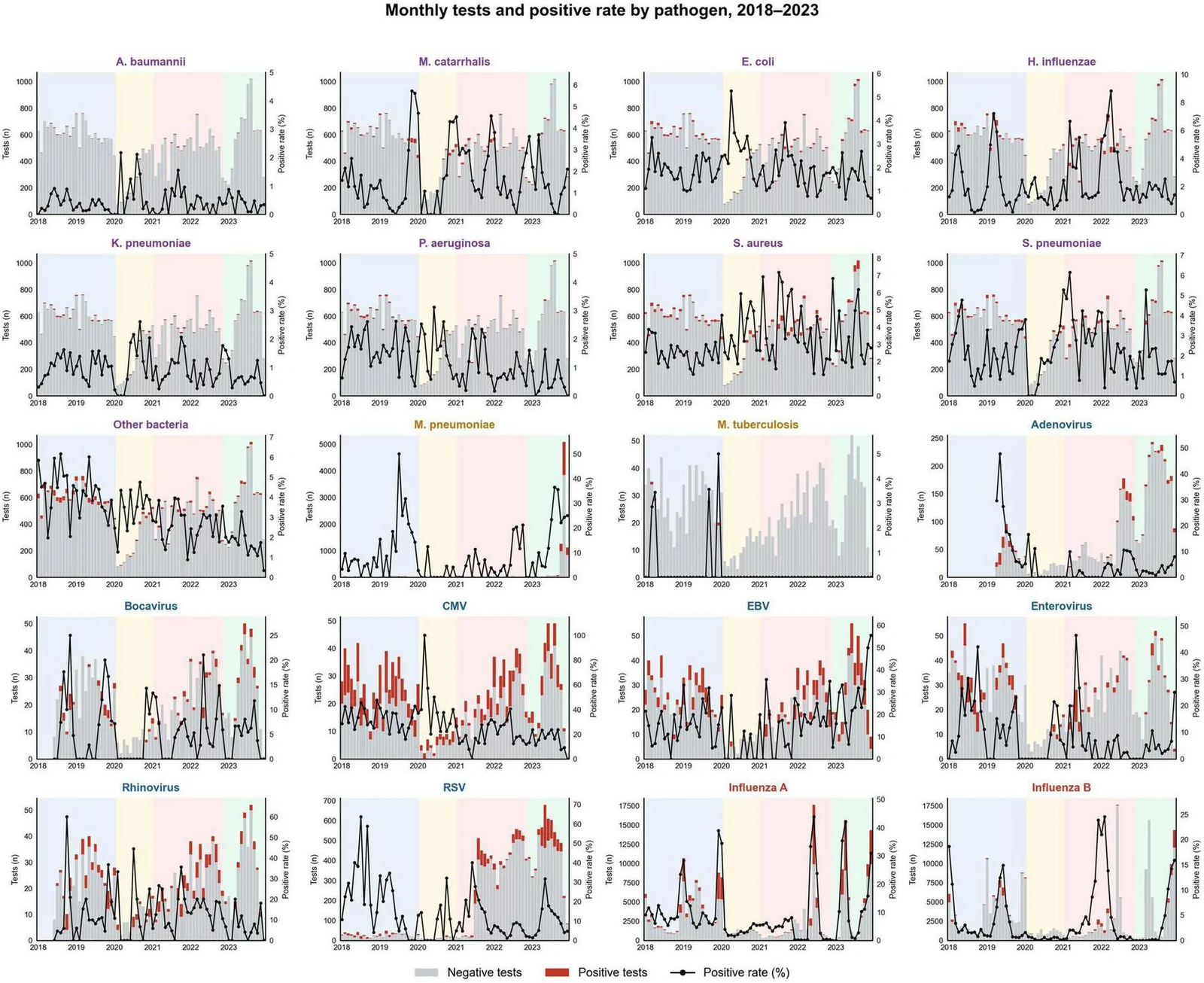
Monthly tests and positive rate for each pathogen, 2018–2023. Stacked bars show the monthly number of tests (gray = negative, red = positive; left axis); the black line is the positive rate (%; right axis). Shaded bands mark the pre-pandemic (blue), Pandemic-I (yellow), Pandemic-II (red) and post-pandemic (green) periods. Axes are scaled per panel.

### 3.2 Pathogen positivity rates

The positivity rates of all 16 pathogens exhibited divergent trajectories across the three pandemic periods (Figure 1 andTable 2). During Pandemic-I, positivity rates declined sharply for most respiratory viruses, with notable exceptions such as rhinovirus, CMV, and bocavirus. Influenza A fell from 19.2% pre-pandemic to 5.0%; influenza B from 6.5% to 0.5%; RSV from 21.8% to 12.3%; and adenovirus from 18.4% to 1.1%. *M. pneumoniae* decreased from 12.4% to 2.2%. During Pandemic-II, influenza A showed increased positivity to 25.0%, already exceeding its pre-pandemic level, while RSV (8.9%), influenza B (6.2%), and *M. pneumoniae* (7.4%) remained below or near baseline. In the post-pandemic period, influenza A continued its ascent to 29.7%; *M. pneumoniae* surged dramatically to 24.1%–the highest recorded rate across all periods; RSV recovered to 12.8% but remained below baseline; and influenza B reached 7.8%, approximating pre-pandemic levels (Figure 1 and Table 2).

**TABLE 2:** Positive rate (*n*/tested, %, 95% CI) by pathogen and pandemic period.

Group	Pathogen	Pre-pandemic	Pandemic-I	Pandemic-II	Post-pandemic
Bacteria	*A. baumannii*	52/14051 (0.4, 0.3–0.5)	22/3245 (0.7, 0.4–1.0)	50/10537 (0.5, 0.4–0.6)	25/7292 (0.3, 0.2–0.5)
	*M. catarrhalis*	232/14051 (1.7, 1.5–1.9)	81/3245 (2.5, 2.0–3.1)	206/10537 (2.0, 1.7–2.2)	96/7292 (1.3, 1.1–1.6)
	*E. coli*	265/14051 (1.9, 1.7–2.1)	83/3245 (2.6, 2.1–3.2)	193/10537 (1.8, 1.6–2.1)	117/7292 (1.6, 1.3–1.9)
	*H. influenzae*	330/14051 (2.3, 2.1–2.6)	45/3245 (1.4, 1.0–1.9)	367/10537 (3.5, 3.1–3.9)	121/7292 (1.7, 1.4–2.0)
	*K. pneumoniae*	145/14051 (1.0, 0.9–1.2)	40/3245 (1.2, 0.9–1.7)	85/10537 (0.8, 0.7–1.0)	44/7292 (0.6, 0.4–0.8)
	*P. aeruginosa*	228/14051 (1.6, 1.4–1.8)	49/3245 (1.5, 1.1–2.0)	108/10537 (1.0, 0.8–1.2)	46/7292 (0.6, 0.5–0.8)
	*S. aureus*	375/14051 (2.7, 2.4–2.9)	135/3245 (4.2, 3.5–4.9)	424/10537 (4.0, 3.7–4.4)	263/7292 (3.6, 3.2–4.1)
	*S. pneumoniae*	336/14051 (2.4, 2.2–2.7)	82/3245 (2.5, 2.0–3.1)	318/10537 (3.0, 2.7–3.4)	137/7292 (1.9, 1.6–2.2)
	Other bacteria	616/14051 (4.4, 4.1–4.7)	126/3245 (3.9, 3.3–4.6)	275/10537 (2.6, 2.3–2.9)	127/7292 (1.7, 1.5–2.1)
Atypical	*M. pneumoniae*	103/828 (12.4, 10.4–14.9)	4/182 (2.2, 0.9–5.5)	48/652 (7.4, 5.6–9.6)	1868/7739 (24.1, 23.2–25.1)
	*M. tuberculosis*	4/698 (0.6, 0.2–1.5)	0/133 (0.0, 0.0–2.8)	0/534 (0.0, 0.0–0.7)	0/338 (0.0, 0.0–1.1)
Virus	Adenovirus	90/489 (18.4, 15.2–22.1)	2/178 (1.1, 0.3–4.0)	72/1356 (5.3, 4.2–6.6)	61/2097 (2.9, 2.3–3.7)
	Bocavirus	28/454 (6.2, 4.3–8.8)	4/94 (4.3, 1.7–10.4)	26/484 (5.4, 3.7–7.8)	19/321 (5.9, 3.8–9.1)
	CMV	213/642 (33.2, 29.6–36.9)	34/92 (37.0, 27.8–47.2)	110/490 (22.4, 19.0–26.3)	58/322 (18.0, 14.2–22.6)
	EBV	102/638 (16.0, 13.3–19.0)	7/90 (7.8, 3.8–15.2)	86/489 (17.6, 14.5–21.2)	89/369 (24.1, 20.0–28.7)
	Enterovirus	94/787 (11.9, 9.9–14.4)	8/119 (6.7, 3.4–12.7)	36/576 (6.2, 4.5–8.5)	16/348 (4.6, 2.8–7.3)
	Rhinovirus	57/468 (12.2, 9.5–15.5)	14/114 (12.3, 7.5–19.6)	76/508 (15.0, 12.1–18.3)	21/328 (6.4, 4.2–9.6)
	RSV	149/682 (21.8, 18.9–25.1)	18/146 (12.3, 7.9–18.6)	643/7240 (8.9, 8.2–9.6)	743/5790 (12.8, 12.0–13.7)
Influenza	Influenza A	14970/78115 (19.2, 18.9–19.4)	623/12508 (5.0, 4.6–5.4)	10426/41735 (25.0, 24.6–25.4)	16770/56467 (29.7, 29.3–30.1)
	Influenza B	5088/78123 (6.5, 6.3–6.7)	63/12508 (0.5, 0.4–0.6)	2601/41736 (6.2, 6.0–6.5)	4432/56466 (7.8, 7.6–8.1)

Denominator = patients tested for that pathogen in that period (bacteria: any culture). 95% CI by Wilson method. Testing populations differ markedly by pathogen and shifted over time (e.g., RSV and Mp moved from selective to routine panel testing, tested-n rising ∼10-fold), so within-pathogen trends and cross-pathogen comparisons of raw rates should be interpreted with this in mind.

In contrast to viruses, bacterial positivity rates showed more modest fluctuations (Table 2). These bacterial trends should be interpreted cautiously because specimen type, hospitalization rates, antibiotic exposure, and culture indications changed over the study period; specimen-specific denominators are provided in Supplementary Table 16. *S. pneumoniae* remained relatively stable across periods (2.4% pre-pandemic, 2.5% in Pandemic-I, 3.0% in Pandemic-II, and 1.9% post-pandemic). *H. influenzae* rose from 2.3% pre-pandemic to 3.5% during Pandemic-II before declining to 1.7% post-pandemic. *S. aureus* increased from 2.7% pre-pandemic to 4.2% in Pandemic-I and remained elevated at 3.6% post-pandemic. *P. aeruginosa* steadily declined from 1.6% pre-pandemic to 0.6% post-pandemic. This overall pattern, characterized by marked viral disruption and relatively stable bacterial rates, was further illustrated in the monthly trend analysis (Figure 1) and the heatmap visualization (Supplementary Figure 2, left panel).

### 3.3 Adjusted odds ratios of pathogen positivity

To account for potential confounding by age, sex, care setting, and hospital campus, we estimated adjusted odds ratios (aOR) for pathogen positivity in each pandemic period relative to pre-pandemic (Figure 2 and Table 3). The primary bacterial model excluded specimen type because it is a near-deterministic mediator of culture yield that shifted markedly across periods; a sensitivity model including specimen type is provided in Supplementary Table 12. For the four most-disrupted respiratory pathogens, the aOR estimates revealed distinct patterns. Influenza A showed a strong suppression during Pandemic-I (aOR = 0.23, 95% CI: 0.21–0.25, *p* < 0.001), followed by a significant increase in positivity in Pandemic-II (aOR = 1.26, 95% CI: 1.23–1.30, *p* < 0.001) and a continued elevation in the post-pandemic period (aOR = 1.52, 95% CI: 1.48–1.56, *p* < 0.001). *M. pneumoniae* exhibited a delayed but dramatic resurgence, with aOR increasing from 0.54 (95% CI: 0.37–0.78, *p* = 0.001) in Pandemic-II to 1.99 (95% CI: 1.54–2.57, *p* < 0.001) post-pandemic. Notably, the adjusted estimates for RSV diverged from the crude rates: although the crude positivity remained below baseline (Table 2), the aOR indicated a significant increase in the post-pandemic period (aOR = 1.31, 95% CI: 1.06–1.63, *p* = 0.012), highlighting the importance of case-mix adjustment given the broadening of RSV testing over time. A similar divergence was observed for *M. pneumoniae*, for which the crude positivity rate increased from 12.4% pre-pandemic to 24.1% post-pandemic despite a marked expansion in testing; the adjusted OR was even higher (aOR = 1.99 overall; aOR = 3.32 in 5–9 year-olds), likely reflecting case-mix changes and denominator inflation due to broadening of testing. Influenza B remained suppressed through Pandemic-II (aOR = 0.84, 95% CI: 0.80–0.88, *p* < 0.001) and only approached baseline in the post-pandemic period (aOR = 0.95, 95% CI: 0.91–1.00, *p* = 0.031; Figure 2 and Table 3).

**FIGURE 2:**
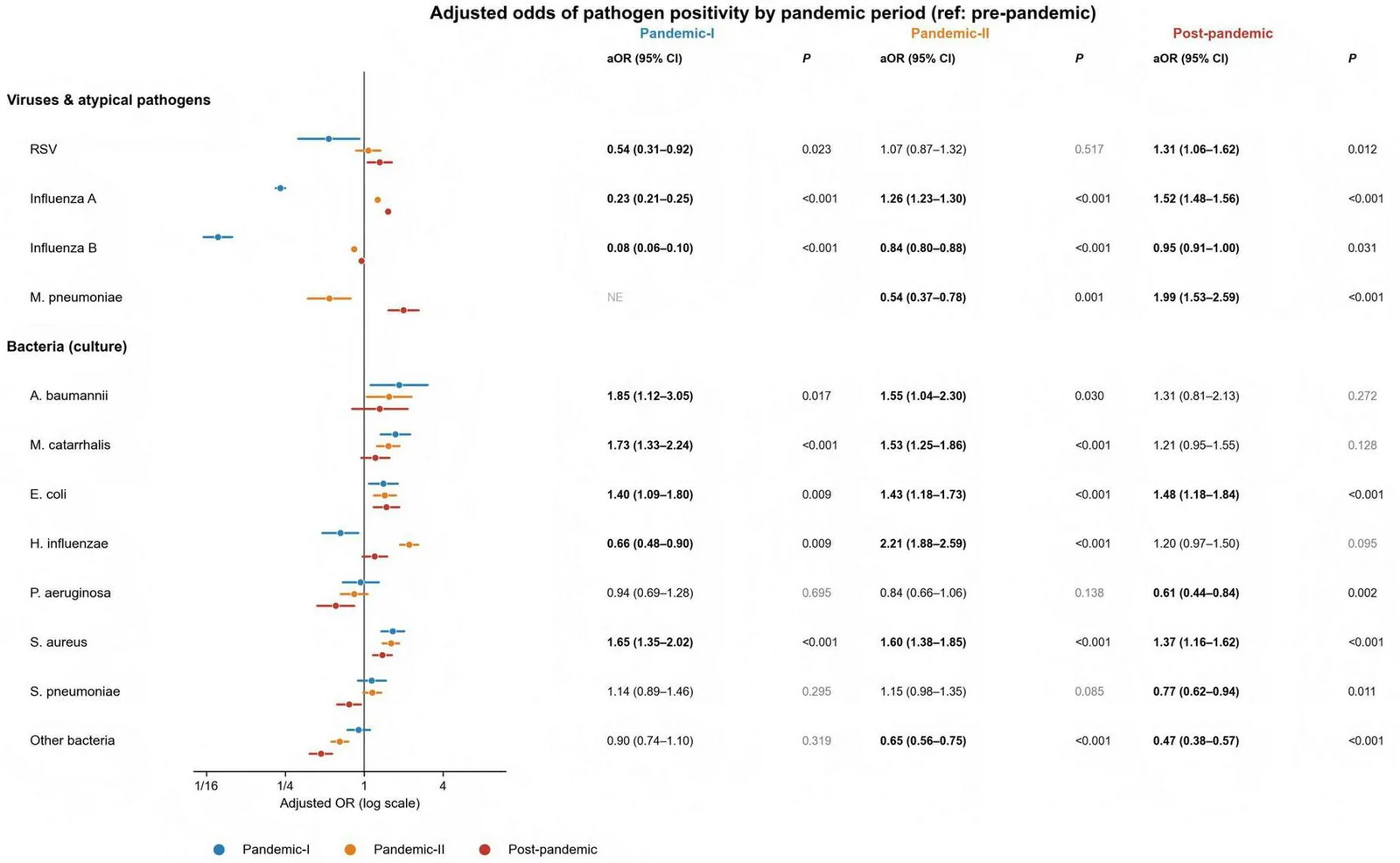
Adjusted odds ratios (aOR) of pathogen positivity in each pandemic period versus pre-pandemic, for the four most-disrupted pathogens and the bacteria significant in ≥1 period. Multivariable logistic regression adjusted for age, sex, care setting, and hospital campus. For bacterial culture analyses, the primary model excluded specimen type as a near-deterministic mediator of culture yield; a sensitivity model including specimen type is provided in Supplementary Table 12. Bars are 95% CIs (log scale); arrowheads indicate CIs beyond the axis. The aOR (95% CI) and *P-*value for each period are tabulated at right; significant aOR (*P* < 0.05) are shown in bold. NE, not estimable.

**TABLE 3:** Adjusted odds ratio of positivity, each pandemic period vs. pre-pandemic, for the disrupted pathogens.

Pathogen	Pandemic-I aOR (95% CI)	*P*	Pandemic-II aOR (95% CI)	*P*	Post aOR (95% CI)	*P*
RSV	0.54 (0.31–0.92)	0.027	1.07 (0.87–1.32)	0.522	1.31 (1.06–1.62)	0.014
Influenza A	0.23 (0.21–0.25)	<0.001	1.26 (1.23–1.30)	<0.001	1.52 (1.48–1.56)	<0.001
Influenza B	0.08 (0.06–0.10)	<0.001	0.84 (0.80–0.88)	<0.001	0.95 (0.91–1.00)	0.025
*M. pneumoniae*	–		0.54 (0.37–0.78)	0.001	1.99 (1.53–2.59)	<0.001
*A. baumannii*	1.85 (1.12–3.05)	0.017	1.55 (1.04–2.30)	0.031	1.31 (0.81–2.13)	0.275
*M. catarrhalis*	1.73 (1.33–2.24)	<0.001	1.53 (1.25–1.86)	<0.001	1.21 (0.95–1.55)	0.131
*E. coli*	1.40 (1.09–1.80)	0.009	1.43 (1.18–1.73)	<0.001	1.48 (1.18–1.84)	<0.001
*H. influenzae*	0.66 (0.48–0.90)	0.009	2.21 (1.88–2.59)	<0.001	1.20 (0.97–1.50)	0.093
*P. aeruginosa*	0.94 (0.69–1.28)	0.696	0.84 (0.66–1.06)	0.140	0.61 (0.44–0.84)	0.002
*S. aureus*	1.65 (1.35–2.02)	<0.001	1.60 (1.38–1.85)	<0.001	1.37 (1.16–1.62)	<0.001
*S. pneumoniae*	1.14 (0.89–1.46)	0.294	1.15 (0.98–1.35)	0.079	0.77 (0.62–0.94)	0.012
Other bacteria	0.90 (0.74–1.10)	0.319	0.65 (0.56–0.75)	<0.001	0.47 (0.38–0.57)	<0.001

Adjusted for age, sex, care setting and hospital campus, with cluster-robust standard errors by child (repeat visits); specimen type excluded as an over-adjusting mediator of culture yield (see specimen sensitivity table). Bacteria shown had an adjusted *P* < 0.05 in ≥1 period. Reference: pre-pandemic. Adjusted ORs may diverge from crude ORs because the tested population broadened over the study (e.g., RSV, Mp); the adjusted estimate is the case-mix-controlled effect.

Among bacteria with at least one period of significant aOR, *S. aureus* consistently showed elevated odds across all three pandemic periods (Pandemic-I aOR = 1.65, Pandemic-II aOR = 1.60, post-pandemic aOR = 1.37; all *p* < 0.001). These estimates may reflect changes in healthcare exposure and infection prevention practices rather than NPI effects alone. *E. coli* also displayed significantly increased odds in all three periods (aOR range: 1.40–1.48, all *p* ≤ 0.009). In contrast, *P. aeruginosa* showed a progressive decline, reaching aOR = 0.61 (95% CI: 0.44–0.84, *p* = 0.002) in the post-pandemic period. For *P. aeruginosa*, the direction of the association reversed after adjustment for specimen type (post-pandemic aOR 0.61 vs. 1.84; Supplementary Table 12), and for *E. coli* the estimate changed substantially (1.48 vs. 4.71). These estimates should therefore be interpreted with caution. *S. pneumoniae* demonstrated a post-pandemic decrease (aOR = 0.77, 95% CI: 0.62–0.94, *p* = 0.011; Figure 2 and Table 3). Of note, the direction of aOR for RSV and several bacteria differed from their crude OR counterparts (Supplementary Table 6). Detailed univariable and multivariable logistic regression results for RSV, influenza A, influenza B, and *M. pneumoniae* are provided in Supplementary Tables 7–10. In a sensitivity analysis restricted to inpatients with more consistent testing indications, the main trends for influenza A and *M. pneumoniae* remained robust, although effect sizes were attenuated (Supplementary Table 11). Comparable-denominator sensitivity analyses further confirmed that the post-pandemic increases in RSV, influenza A, and *M. pneumoniae* persisted as the denominator was progressively restricted (Supplementary Figure 3). For influenza B, the inpatient sensitivity analysis showed persistent suppression across all periods (Pandemic-II aOR = 0.43, 95% CI: 0.26–0.69; post-pandemic aOR = 0.15, 95% CI: 0.06–0.34; Supplementary Table 11). In contrast, the overall post-pandemic estimate for influenza B was 0.95 (95% CI 0.91–1.00), and the estimate among adolescents was 2.88 (95% CI 2.51–3.30). The discrepancy between the inpatient estimate (0.15) and the overall/adolescent estimates suggests that the adolescent influenza B resurgence was predominantly an outpatient phenomenon. Notably, in the inpatient sensitivity analysis, influenza A positivity during Pandemic-II was suppressed (aOR = 0.64, 95% CI: 0.44–0.95), in contrast to the elevated estimate in the full cohort (aOR = 1.26, 95% CI: 1.23–1.30), suggesting that the early rebound in influenza A was driven primarily by outpatient testing.

### 3.4 Age-stratified analysis of the four most-disrupted pathogens

To further characterize the heterogeneous impact of the pandemic across age groups, we performed age-stratified analyses for RSV, influenza A, influenza B, and *M. pneumoniae* (Figure 3 and Table 4). Significant age–period interactions were observed for all four pathogens (*p* < 0.001 for each), indicating that the pandemic effects varied markedly by age.

**FIGURE 3:**
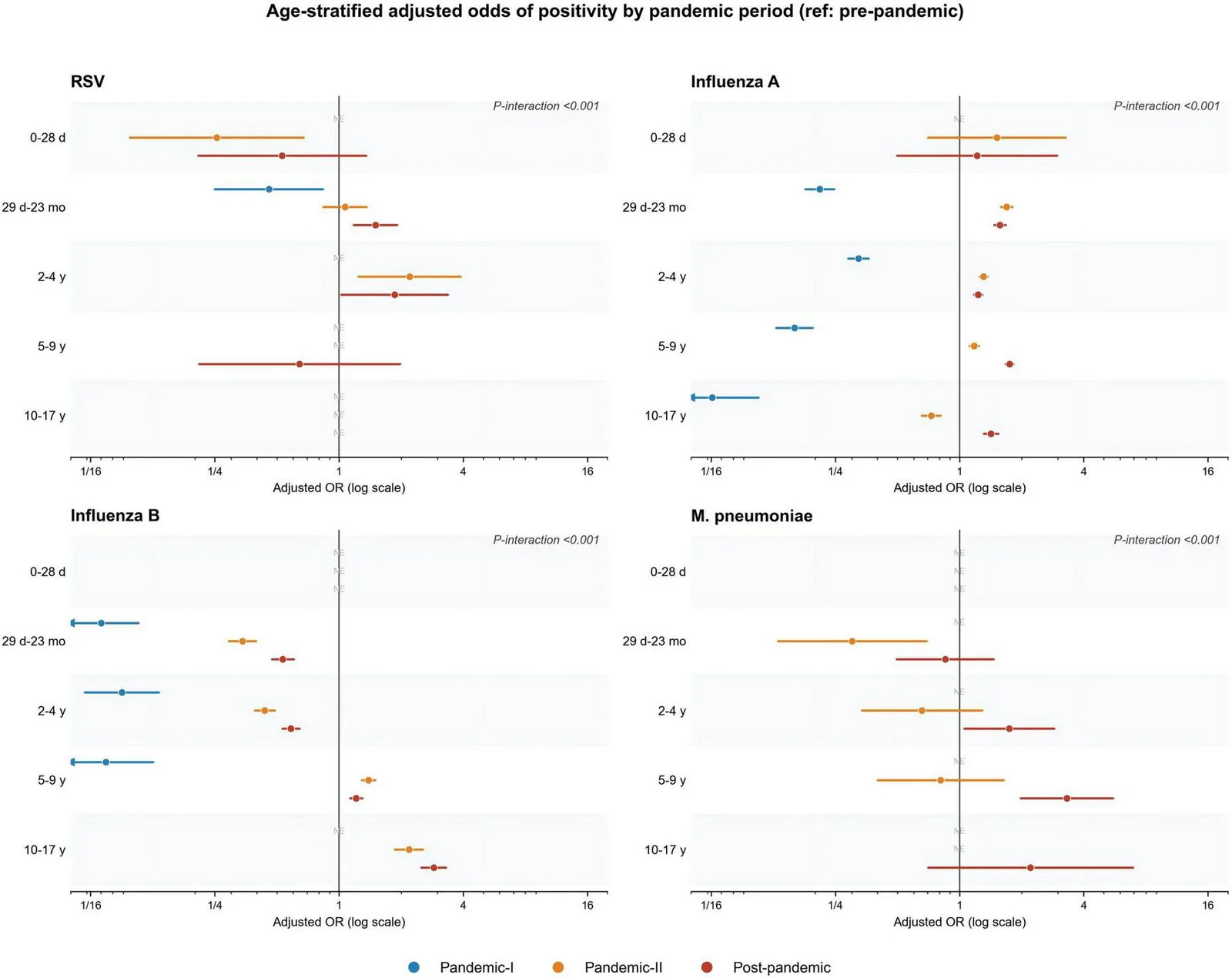
Age-stratified adjusted odds of positivity by pandemic period (reference pre-pandemic) for RSV, influenza A, influenza B and *M. pneumoniae*. Adjusted within each age stratum for sex, care setting and hospital campus. *P*-interaction = robust Wald test for age × period interaction based on cluster-robust variance estimates.

**TABLE 4:** Age-stratified adjusted OR (each period vs. pre-pandemic) for the four most-disrupted pathogens.

Pathogen	Age group	Pandemic-I aOR (95% CI)	Pandemic-II aOR (95% CI)	Post aOR (95% CI)
RSV	0–28 d	NE	0.26 (0.09–0.77)*	0.53 (0.18–1.52)
	29 d–23 mo	0.46 (0.25–0.84)*	1.07 (0.84–1.36)	1.50 (1.18–1.91)**
	2–4 y	NE	2.20 (1.23–3.92)**	1.86 (1.00–3.45)*
	5–9 y	NE	NE	NE
	10–17 y	NE	NE	NE
Influenza A	0–28 d	NE	1.52 (0.71–3.22)	1.22 (0.50–2.98)
	29 d–23 mo	0.21 (0.18–0.25)***	1.69 (1.59–1.80)***	1.57 (1.47–1.67)***
	2–4 y	0.32 (0.29–0.36)***	1.31 (1.25–1.37)***	1.23 (1.17–1.28)***
	5–9 y	0.16 (0.13–0.19)***	1.18 (1.12–1.24)***	1.74 (1.67–1.82)***
	10–17 y	0.06 (0.04–0.11)***	0.73 (0.66–0.81)***	1.42 (1.31–1.54)***
Influenza B	0–28 d	NE	NE	NE
	29 d–23 mo	0.07 (0.05–0.11)***	0.34 (0.29–0.40)***	0.53 (0.47–0.60)***
	2–4 y	0.09 (0.06–0.13)***	0.44 (0.39–0.49)***	0.58 (0.53–0.64)***
	5–9 y	0.07 (0.04–0.13)***	1.39 (1.29–1.50)***	1.21 (1.13–1.30)***
	10–17 y	NE	2.18 (1.87–2.55)***	2.88 (2.51–3.30)***
*M. pneumoniae*	0–28 d	NE	NE	NE
	29 d–23 mo	NE	0.30 (0.13–0.69)**	0.85 (0.51–1.44)
	2–4 y	NE	0.66 (0.34–1.29)	1.74 (1.07–2.84)*
	5–9 y	NE	0.81 (0.40–1.65)	3.32 (1.98–5.55)***
	10–17 y	NE	NE	2.21 (0.73–6.66)

Adjusted within each age stratum for sex, care setting and hospital campus. NE, not estimable (<5 events or 95% CI width >10-fold).

*P < 0.05,

**P < 0.01,

***P < 0.001. Age × period interaction *P*-values were obtained using robust Wald tests based on cluster-robust variance estimates; *P* < 0.001 for all four pathogens.

Influenza A showed consistent post-pandemic increases in positivity across all age groups except infants aged 0–28 days. The strongest change in positivities were observed in school-aged children (5–9 years; post-pandemic aOR = 1.74, 95% CI: 1.67–1.82) and adolescents (10–17 years; post-pandemic aOR = 1.42, 95% CI: 1.31–1.54). Notably, in adolescents, influenza A remained suppressed during Pandemic-II (aOR = 0.73, 95% CI: 0.66–0.81) before rebounding post-pandemic.

Influenza B exhibited a strikingly different age pattern. In young children (29 days–23 months and 2–4 years), influenza B remained significantly suppressed across all three pandemic periods (post-pandemic aOR = 0.53 and 0.58, respectively; both *p* < 0.001). In contrast, in older children and adolescents (5–9 years and 10–17 years), influenza B showed a strong increase in positivity in the post-pandemic period (aOR = 1.21 and 2.88, respectively; both *p* < 0.001), with adolescents showing the most dramatic increase.

*M. pneumoniae* displayed a delayed and age-restricted resurgence. The post-pandemic increase in positivity was most pronounced in school-aged children (5–9 years; aOR = 3.32, 95% CI: 1.98–5.55, *p* < 0.001), with moderate increases in children aged 2–4 years (aOR = 1.74, 95% CI: 1.07–2.84, *p* = 0.029). No significant post-pandemic increase was observed in infants (29 days–23 months) or adolescents (10–17 years; Figure 3 and Table 4).

RSV showed a post-pandemic increase primarily in young children aged 29 days–23 months (aOR = 1.50, 95% CI: 1.18–1.91, *p* < 0.001) and children aged 2–4 years (aOR = 1.86, 95% CI: 1.00–3.45, *p* = 0.043), whereas no significant change in positivity was observed in school-aged children or adolescents. Infants aged 0–28 days showed suppression during Pandemic-II (aOR = 0.26, 95% CI: 0.09–0.77) with no significant post-pandemic change (Figure 3 and Table 4).

### 3.5 Co-infection patterns

Co-infection patterns shifted across periods (Supplementary Table 4). Influenza A–B dual positivity accounted for approximately half of all co-infections (49.7% overall); however, this may partly reflect rapid-antigen cross-reactivity rather than true dual infection. In a sensitivity analysis excluding influenza A–B dual-antigen positive events, bacterial–influenza co-infections increased substantially in the post-pandemic period, whereas bacterial–bacterial co-infections decreased from 62.0% in Pandemic-I to 11.2% post-pandemic (Supplementary Table 13). Age-stratified analysis revealed that influenza A–B co-infections were most common in children aged 5–9 years (70.8%) and 10–17 years (74.3%; Supplementary Table 5).

## 4 Discussion

In this large-scale, 6-year period-comparison study of pediatric respiratory pathogens in southern China, we documented a profound and pathogen-specific reshaping of the respiratory infectious disease landscape across the COVID-19 pandemic and its aftermath. Our findings can be distilled into three core observations. These patterns should be interpreted in the context of changing testing practices. The expansion of testing, particularly for RSV and *M. pneumoniae*, may have diluted crude positivity rates, whereas the adjusted estimates and comparable-denominator sensitivity analyses suggested that the post-pandemic increases were not solely an artifact of testing expansion.

This issue is particularly relevant for RSV and *M. pneumoniae*, whose testing expanded approximately 8.5-fold and 9.3-fold, respectively, from pre-pandemic to post-pandemic. When testing expansion predominantly includes mildly symptomatic or low-pathogen-load children, crude positivity rates are expected to underestimate true epidemic intensity, whereas testing targeted to severely ill or hospitalized children may enrich for true cases. Crude positivity rates were therefore presented for descriptive purposes only; the primary evidence for cross-period comparisons came from adjusted odds ratios and from sensitivity analyses restricted to comparable-denominator subgroups. For RSV, crude positivity remained below baseline post-pandemic, whereas the adjusted OR was significantly elevated (aOR = 1.31, 95% CI: 1.06–1.63). For *M. pneumoniae*, crude positivity rose from 12.4% pre-pandemic to 24.1% post-pandemic despite a 9.3-fold expansion in testing, and the adjusted OR was even higher (aOR = 1.99 overall; aOR = 3.32 in 5–9 year-olds). In sensitivity analyses restricted to inpatients, in whom testing is more consistently driven by clinical indications and less affected by routine outpatient screening, the post-pandemic increases in RSV and *M. pneumoniae* remained significant, although effect sizes were attenuated. These findings suggest that the observed increases were not solely an artifact of testing expansion, although residual confounding by changes in inpatient case-mix, specimen type, and testing indications cannot be fully excluded.

First, the hypothesized “immunity gap” may have contributed to the observed patterns, but this remains speculative because no serological or immunological data were collected. While most viruses and *M. pneumoniae* were suppressed during the stringent NPI period (Pandemic-I), their post-pandemic changes in positivity were far from uniform. Influenza A increased positivity across all pediatric ages and exceeded baseline levels as early as Pandemic-II. This pattern may be partially explained by its high transmissibility and short serial interval (Cowling et al., 2009, 2020), although this hypothesis cannot be tested with the current data. In contrast, *M. pneumoniae* showed the most delayed but most pronounced increase in positivity (aOR = 1.99 overall; aOR = 3.32 in 5–9 year-olds). One possible explanation is its longer incubation period, slower transmission dynamics, and the accumulation of a large susceptible pool during the prolonged suppression period (Liu et al., 2024; Meyer Sauteur and Beeton, 2024), but this remains speculative. RSV showed a moderate and selective increase in positivity in young children (the age group with the highest baseline susceptibility) supporting the hypothesis that would require serological and vaccination data to confirm is that waning maternal antibodies and lack of natural boosting during the pandemic created a concentrated “susceptible cohort” in the 0–4 year age range (Billard and Bont, 2023; Bont et al., 2016). Influenza B never recovered in young children but surged in adolescents, suggesting age-specific immunological or behavioral factors that warrant further investigation. These findings align with recent reports documenting pathogen-specific changes in positivity in other regions (Chow et al., 2023).

Second, the age–period interaction was statistically significant for all four major pathogens (RSV, influenza A, influenza B, and *M. pneumoniae*), underscoring age as a fundamental effect modifier rather than merely a confounder. This implies that a one-size-fits-all post-pandemic surveillance strategy would be inadequate; instead, surveillance may need to be stratified by both pathogen and age. The biological plausibility of this age dependency is supported by age-related differences in pre-existing immunity, social mixing patterns, and physiological susceptibility to specific pathogens.

Third, bacterial pathogens exhibited a fundamentally different dynamic. The divergent bacterial trends observed in our study-persistently elevated *S. aureus* and *E. coli* positivity alongside declining *S. pneumoniae* and *P. aeruginosa*-should be interpreted cautiously, given the potential influence of shifting specimen types, hospitalization rates, antibiotic exposure, and culture indications. Specimen type lies on the causal pathway between pandemic period and culture positivity: the proportion of upper-respiratory specimens increased from 91.9% pre-pandemic to 99.2% post-pandemic, and lower-respiratory specimens yielded substantially higher culture positivity (e.g., *P. aeruginosa* 13.6×, *E. coli* 8.7×; Supplementary Table 16). Because specimen type lies on the causal pathway between pandemic period and culture positivity, we consider the primary model (without specimen type) to be the more appropriate primary analysis, while presenting the specimen-type-adjusted model in Supplementary Table 12 for transparency. However, we acknowledge that neither estimate should be regarded as definitively preferable. Similar challenges in attributing bacterial trends to specific causes have been highlighted in recent reviews of severe bacterial infections, which emphasize the importance of antimicrobial stewardship and local resistance surveillance in interpreting such patterns (Maniaci et al., 2026). Unlike viruses, which were suppressed by NPIs and showed changes in positivity post-pandemic, *S. aureus* and *E. coli* were persistently elevated across all pandemic periods. This pattern may potentially reflect increased healthcare exposure rather than NPI effects; this hypothesis cannot be tested with the current data, given the higher proportion of inpatients during the pandemic periods (Weiner-Lastinger et al., 2022). These divergent bacterial trends may reflect changes in healthcare exposure, antibiotic prescribing, infection control practices, and culture indications, and should not be attributed solely to NPIs. Conversely, *S. pneumoniae* and *P. aeruginosa* declined post-pandemic, which may reflect changes in antibiotic prescribing, infection control practices, or altered host–pathogen ecological interactions (Baker et al., 2020; Maniaci et al., 2026). These observations reinforce the need for continued antimicrobial stewardship and pathogen-specific surveillance, particularly as the post-pandemic era may bring unpredictable alternations in bacterial and viral dominance (Baker et al., 2020; Maniaci et al., 2026). Beyond these direct bacterial trends, the shifting co-infection patterns across periods provide further insights into pathogen ecological interactions. The dominance of bacterial–bacterial co-infections during Pandemic-I (49.0%) coincided with the nadir of viral circulation. This suggests that in the absence of viral co-pathogens, bacterial–bacterial interactions became relatively more prominent, possibly driven by healthcare setting exposure. The post-pandemic surge in bacterial–influenza co-infections (22.9%) aligns with the well-established synergism between influenza and bacterial superinfection, particularly with *S. pneumoniae* and *S. aureus* (Mina and Klugman, 2014; Morens et al., 2008). It should also be noted that CMV and EBV detection may reflect reactivation rather than acute respiratory infection, and should therefore be interpreted separately from acute respiratory pathogens. Due to unequal period lengths, this study is a period comparison rather than a strict time-series model; future studies should employ interrupted time-series methods.

Our findings are broadly consistent with international reports documenting the suppression of respiratory viruses during early COVID-19 and their subsequent change in positivity (Britton et al., 2020; Huang et al., 2021; Kuitunen et al., 2020; Leija-Martínez et al., 2024), as well as with recent syntheses emphasizing the profound impact of COVID-19 non-pharmaceutical interventions (NPIs) on respiratory pathogen circulation and the often heterogeneous changes in infection patterns after their relaxation (Guarino et al., 2026). As reported in other settings, the suppression of influenza and RSV during strict NPI periods was followed by a resurgence that varied by pathogen, age, and timing (Chow et al., 2023; Guarino et al., 2026). However, our study extends the literature by including a comprehensive panel of 16 pathogens, systematically quantifying the age-modifying effect, and covering the full transition from strict NPIs through relaxation to complete reopening. Notably, the exceptionally high post-pandemic increase in positivity of *M. pneumoniae* (aOR = 1.99 overall; aOR = 3.32 in 5–9 year-olds) exceeds most previously reported estimates (Beeton et al., 2020; Liu et al., 2024), likely reflecting the prolonged absence of this pathogen and the large susceptible child population in the Guangzhou region. This delayed but marked increase, particularly among school-aged children, may be consistent with the immunity gap hypothesis (Cohen et al., 2021; Guarino et al., 2026; Messacar et al., 2022), although serological data were not available in our study to confirm this mechanism. At the same time, the post-pandemic landscape should not be viewed as a simple return to baseline; rather, it reflects complex interactions among waning population immunity, altered pathogen ecology, and changes in healthcare-seeking and testing practices (Chow et al., 2023; Guarino et al., 2026). The discrepancies between inpatient and overall estimates for influenza A and B further suggest that outpatient testing contributed substantially to the observed patterns, particularly for the early influenza A rebound during Pandemic-II and the adolescent influenza B resurgence post-pandemic. Our age-stratified findings further demonstrate that these changes are not uniform across pediatric age groups, supporting the need for age- and pathogen-specific surveillance.

Our findings carry several actionable implications. First, age- and pathogen-specific surveillance systems may warrant consideration, disaggregating data by age strata and pathogen groups. Second, vaccination strategies could be considered for recalibration, particularly ensuring high influenza vaccine coverage in adolescents. Third, clinical preparedness may need to anticipate that future seasons may bring unpredictable alternation of pathogen dominance. Fourth, the sustained elevation of *S. aureus* and *E. coli* may warrant attention with respect to healthcare-associated infections, warranting enhanced infection prevention efforts.

## 5 Limitations

This study has several limitations. The primary limitation is testing bias: test positivity among tested children is not equivalent to community incidence. Denominators changed markedly over time, especially for RSV and *M. pneumoniae*, and testing indications may have shifted. Testing indications were not systematically recorded in the source database; inpatient status was therefore used as a proxy for clinically indicated testing. Expansion of testing may introduce many mildly symptomatic or asymptomatic children with low pathogen load, thereby diluting crude positivity rates, whereas targeted testing in inpatients may enrich for true cases. Crude positivity rates are therefore presented for descriptive purposes only, whereas adjusted odds ratios and comparable-denominator sensitivity analyses provide the primary evidence. In sensitivity analyses restricted to inpatients, in whom testing is more consistently driven by clinical indications and less affected by routine outpatient screening, the post-pandemic increases in RSV and *M. pneumoniae* remained significant, although effect sizes were attenuated. This supports the interpretation that the observed increases were not solely an artifact of outpatient testing expansion, although residual confounding by changes in inpatient case-mix, specimen type, and testing indications cannot be fully excluded. Second, the retrospective, single-region design limits generalizability. Third, although adjusted analyses controlled for measured confounders, residual confounding by unmeasured testing indications may remain. Fourth, influenza A and B were detected by rapid antigen testing, which has lower sensitivity than PCR. Although the same platform was used across all periods and the main trends persisted in inpatient sensitivity analyses, we cannot fully exclude misclassification due to antigen sensitivity. The influenza A–B dual positivity category may also be inflated by rapid antigen cross-reactivity. Fifth, bacterial culture positivity rates are influenced by prior antibiotic use and specimen quality. Finally, some age–period–pathogen strata had sparse events (denoted “NE” in Table 4). The inpatient sensitivity analysis for influenza B was based on a small number of events and should be interpreted with caution.

## 6 Conclusion

The COVID-19 pandemic fundamentally disrupted the epidemiology of pediatric respiratory pathogens in southern China, but the post-pandemic landscape is not a simple return to the pre-pandemic baseline. Rather, it is characterized by heterogeneous, pathogen-specific, and age-dependent changes in test positivity. Influenza A showed increased positivity across all pediatric ages; RSV showed a selective increase in young children; *M. pneumoniae* exhibited a delayed but marked increase in school-aged children; Influenza B showed a bimodal pattern, with persistent suppression in young children and marked resurgence in adolescents. Bacterial pathogens showed divergent trends, with some (*S. aureus*, *E. coli*) persistently elevated and others (*S. pneumoniae*, *P. aeruginosa*) declining. These findings may support consideration of age-stratified surveillance and tailored clinical preparedness in the post-pandemic era.

## Data Availability

The original contributions presented in this study are included in this article/Supplementary material, further inquiries can be directed to the corresponding author.

## Footnotes

1
http://gd.people.com.cn/n2/2020/0123/c123932-33742154.html

## Supplementary material

Supplementary Figure 1 Respiratory pathogen test records were aggregated to patient-periods (one record per child per pandemic period) and classified by testing modality.

Supplementary Figure 2 Left panel: positive rate (%) of each pathogen by pandemic period (color on a square-root scale). Right panel: crude odds ratio of positivity in each pandemic period versus pre-pandemic (blue < 1 = suppression, red > 1 = change in positivity); **P* < 0.05, ***P* < 0.01, ****P* < 0.001; NE = not estimable. Denominators differ markedly by pathogen and shifted over time (RSV and Mp moved from selective to routine testing); for RSV and several bacteria the case-mix-adjusted direction (Figure 2) differs from the crude rate/OR shown here.

Supplementary Figure 3 Comparable-denominator sensitivity. Adjusted odds ratios (period vs. pre-pandemic) for the four key pathogens under progressively more stable testing indications: full cohort, inpatients, and inpatients with lower-respiratory specimens. The post-pandemic rebound of RSV, influenza A and *M. pneumoniae* persists (and strengthens for RSV/Mp) as the denominator is restricted to clinically indicated testing, showing it is not an artifact of the ∼10-fold denominator expansion. Adjusted for age, sex and hospital campus with cluster-robust standard errors by child; NE = not estimable (sparse).

Supplementary Table 1 Baseline characteristics by pandemic period (patients with bacterial culture).

Supplementary Table 2 Baseline characteristics by pandemic period (patients with viral PCR).

Supplementary Table 3 Baseline characteristics by pandemic period (patients with influenza antigen test).

Supplementary Table 4 Co-infection patterns by pandemic period.

Supplementary Table 5 Co-infection patterns by age group.

Supplementary Table 6 Crude odds ratio of positivity, each pandemic period vs. pre-pandemic, by pathogen.

Supplementary Table 7 Univariable and multivariable logistic regression for RSV positivity.

Supplementary Table 8 Univariable and multivariable logistic regression for Influenza A positivity.

Supplementary Table 9 Univariable and multivariable logistic regression for Influenza B positivity.

Supplementary Table 10 Univariable and multivariable logistic regression for *M. pneumoniae* positivity.

Supplementary Table 11 Sensitivity-adjusted OR restricted to inpatients (more consistent testing indications).

Supplementary Table 12 Sensitivity - bacterial adjusted OR with vs. without specimen type in the model.

Supplementary Table 13 Sensitivity-co-infection by period EXCLUDING influenza A-B dual-antigen positivity.

Supplementary Table 14 Number of patients tested per pathogen and period (testing-volume change).

Supplementary Table 15 Comparable-denominator sensitivity-adjusted OR (each period vs. pre-pandemic) under progressively stable testing indications.

Supplementary Table 16 Specimen-specific bacterial positivity (culture yield by specimen type).

Supplementary Table 17 Data dictionary for the analysis dataset.
