# Supplementary material for: Disruption and change in positivity of respiratory pathogen epidemiology in children during and after the COVID-19 pandemic, 2018–2023: a large-scale period-comparison study in Guangzhou, southern China
Source: Front Microbiol. 2026 Oct 20;17:1992791. doi: 10.3389/fmicb.2026.1992791 (PMC13727640; doi:10.3389/fmicb.2026.1992791)
Supplement: Supplementary file 1 [file Table_1.docx]

**Table S1. Baseline characteristics by pandemic period (patients with bacterial culture).**

| **Variable** | **Level** | **Pre-pandemic** | **Pandemic-I** | **Pandemic-II** | **Post-pandemic** | **Overall** | ***P*** |
| --- | --- | --- | --- | --- | --- | --- | --- |
| N (patient-periods) |  | 14,051 | 3,245 | 10,537 | 7,292 | 35,125 |  |
| Hospital campus | Panyu | 7,498 (53.4) | 1,621 (50.0) | 6,306 (59.8) | 4,124 (56.6) | 19,549 (55.7) | <0.001 |
|  | YX | 6,553 (46.6) | 1,624 (50.0) | 4,231 (40.2) | 3,168 (43.4) | 15,576 (44.3) |  |
| Care setting | Inpatient | 13,355 (95.0) | 3,171 (97.7) | 10,437 (99.1) | 7,183 (98.5) | 34,146 (97.2) | <0.001 |
|  | Outpatient | 696 (5.0) | 74 (2.3) | 100 (0.9) | 109 (1.5) | 979 (2.8) |  |
| Sex | Female | 5,189 (36.9) | 1,255 (38.7) | 4,100 (38.9) | 2,856 (39.2) | 13,400 (38.1) | 0.002 |
|  | Male | 8,862 (63.1) | 1,990 (61.3) | 6,437 (61.1) | 4,436 (60.8) | 21,725 (61.9) |  |
| Specimen | Upper resp. | 6,901 (49.1) | 1,475 (45.5) | 6,613 (62.8) | 6,754 (92.6) | 21,743 (61.9) | <0.001 |
|  | Lower resp. | 7,150 (50.9) | 1,770 (54.5) | 3,924 (37.2) | 538 (7.4) | 13,382 (38.1) |  |
| Age group | 0-28 d | 2,111 (15.0) | 591 (18.2) | 1,082 (10.3) | 702 (9.6) | 4,486 (12.8) | <0.001 |
|  | 29 d-23 mo | 7,021 (50.0) | 1,530 (47.1) | 3,705 (35.2) | 2,099 (28.8) | 14,355 (40.9) |  |
|  | 2-4 y | 3,244 (23.1) | 837 (25.8) | 3,641 (34.6) | 2,106 (28.9) | 9,828 (28.0) |  |
|  | 5-9 y | 1,470 (10.5) | 252 (7.8) | 1,910 (18.1) | 2,063 (28.3) | 5,695 (16.2) |  |
|  | 10-17 y | 205 (1.5) | 35 (1.1) | 199 (1.9) | 322 (4.4) | 761 (2.2) |  |
| Year | 2018 | 7,085 (50.4) | 0 (0.0) | 0 (0.0) | 0 (0.0) | 7,085 (20.2) | <0.001 |
|  | 2019 | 6,623 (47.1) | 0 (0.0) | 0 (0.0) | 0 (0.0) | 6,623 (18.9) |  |
|  | 2020 | 343 (2.4) | 2,907 (89.6) | 0 (0.0) | 0 (0.0) | 3,250 (9.3) |  |
|  | 2021 | 0 (0.0) | 338 (10.4) | 5,197 (49.3) | 0 (0.0) | 5,535 (15.8) |  |
|  | 2022 | 0 (0.0) | 0 (0.0) | 5,340 (50.7) | 256 (3.5) | 5,596 (15.9) |  |
|  | 2023 | 0 (0.0) | 0 (0.0) | 0 (0.0) | 7,036 (96.5) | 7,036 (20.0) |  |
| Length of stay, d (inpatients) | Median [IQR] | 8 [6–11] | 7 [6–10] | 6 [4–8] | 6 [2–8] | 7 [5–9] | <0.001 |
|  | Inpatients with data, n | 13,355 | 3,171 | 10,437 | 7,183 | 34,146 |  |
| Hospital cost, ¥ (inpatients) | Median [IQR] | 8459 [5169–13583] | 7165 [4174–12138] | 8290 [4161–11337] | 8078 [4596–9997] | 8165 [4617–11669] | <0.001 |
|  | Inpatients with data, n | 13,354 | 3,171 | 10,437 | 7,183 | 34,145 |  |

*Values are n (%) unless stated. P from chi-square (categorical) or Kruskal–Wallis (continuous). Analysis unit: patient-period (one record per patient per period); unique children also shown. Length of stay and hospital cost are restricted to inpatients with observed data (outpatients have no admission).*

**Table S2. Baseline characteristics by pandemic period (patients with viral PCR).**

| **Variable** | **Level** | **Pre-pandemic** | **Pandemic-I** | **Pandemic-II** | **Post-pandemic** | **Overall** | ***P*** |
| --- | --- | --- | --- | --- | --- | --- | --- |
| N (patient-periods) |  | 1,255 | 316 | 7,532 | 13,171 | 22,274 |  |
| Hospital campus | Panyu | 710 (56.6) | 156 (49.4) | 6,399 (85.0) | 6,071 (46.1) | 13,336 (59.9) | <0.001 |
|  | YX | 545 (43.4) | 160 (50.6) | 1,133 (15.0) | 7,100 (53.9) | 8,938 (40.1) |  |
| Care setting | Inpatient | 887 (70.7) | 299 (94.6) | 7,318 (97.2) | 5,751 (43.7) | 14,255 (64.0) | <0.001 |
|  | Outpatient | 368 (29.3) | 17 (5.4) | 214 (2.8) | 7,420 (56.3) | 8,019 (36.0) |  |
| Sex | Female | 462 (36.8) | 112 (35.4) | 3,133 (41.6) | 5,786 (43.9) | 9,493 (42.6) | <0.001 |
|  | Male | 793 (63.2) | 204 (64.6) | 4,399 (58.4) | 7,385 (56.1) | 12,781 (57.4) |  |
| Specimen | Upper resp. | 453 (36.1) | 70 (22.2) | 5,984 (79.4) | 12,759 (96.9) | 19,266 (86.5) | <0.001 |
|  | Lower resp. | 802 (63.9) | 246 (77.8) | 1,548 (20.6) | 412 (3.1) | 3,008 (13.5) |  |
| Age group | 0-28 d | 96 (7.6) | 108 (34.2) | 4,955 (65.8) | 3,308 (25.1) | 8,467 (38.0) | <0.001 |
|  | 29 d-23 mo | 671 (53.5) | 151 (47.8) | 1,461 (19.4) | 2,055 (15.6) | 4,338 (19.5) |  |
|  | 2-4 y | 311 (24.8) | 34 (10.8) | 839 (11.1) | 3,000 (22.8) | 4,184 (18.8) |  |
|  | 5-9 y | 149 (11.9) | 17 (5.4) | 227 (3.0) | 3,693 (28.0) | 4,086 (18.3) |  |
|  | 10-17 y | 28 (2.2) | 6 (1.9) | 50 (0.7) | 1,115 (8.5) | 1,199 (5.4) |  |
| Year | 2018 | 510 (40.6) | 0 (0.0) | 0 (0.0) | 0 (0.0) | 510 (2.3) | <0.001 |
|  | 2019 | 712 (56.7) | 0 (0.0) | 0 (0.0) | 0 (0.0) | 712 (3.2) |  |
|  | 2020 | 33 (2.6) | 290 (91.8) | 0 (0.0) | 0 (0.0) | 323 (1.5) |  |
|  | 2021 | 0 (0.0) | 26 (8.2) | 2,769 (36.8) | 0 (0.0) | 2,795 (12.5) |  |
|  | 2022 | 0 (0.0) | 0 (0.0) | 4,763 (63.2) | 446 (3.4) | 5,209 (23.4) |  |
|  | 2023 | 0 (0.0) | 0 (0.0) | 0 (0.0) | 12,725 (96.6) | 12,725 (57.1) |  |
| Length of stay, d (inpatients) | Median [IQR] | 11 [8–17] | 10 [7–14] | 7 [6–10] | 8 [6–11] | 8 [6–11] | <0.001 |
|  | Inpatients with data, n | 887 | 299 | 7,318 | 5,694 | 14,198 |  |
| Hospital cost, ¥ (inpatients) | Median [IQR] | 20398 [10960–42577] | 12403 [7122–23209] | 6539 [4543–11881] | 6500 [4451–10927] | 6855 [4628–12985] | <0.001 |
|  | Inpatients with data, n | 887 | 299 | 7,318 | 5,751 | 14,255 |  |

*Values are n (%) unless stated. P from chi-square (categorical) or Kruskal–Wallis (continuous). Analysis unit: patient-period (one record per patient per period); unique children also shown. Length of stay and hospital cost are restricted to inpatients with observed data (outpatients have no admission).*

**Table S3. Baseline characteristics by pandemic period (patients with influenza antigen test).**

| **Variable** | **Level** | **Pre-pandemic** | **Pandemic-I** | **Pandemic-II** | **Post-pandemic** | **Overall** | ***P*** |
| --- | --- | --- | --- | --- | --- | --- | --- |
| N (patient-periods) |  | 78,124 | 12,508 | 41,736 | 56,468 | 188,836 |  |
| Hospital campus | Panyu | 49,144 (62.9) | 7,718 (61.7) | 26,708 (64.0) | 36,962 (65.5) | 120,532 (63.8) | <0.001 |
|  | YX | 28,980 (37.1) | 4,790 (38.3) | 15,028 (36.0) | 19,506 (34.5) | 68,304 (36.2) |  |
| Care setting | Inpatient | 4,726 (6.0) | 594 (4.7) | 991 (2.4) | 846 (1.5) | 7,157 (3.8) | <0.001 |
|  | Outpatient | 73,398 (94.0) | 11,914 (95.3) | 40,745 (97.6) | 55,622 (98.5) | 181,679 (96.2) |  |
| Sex | Female | 33,732 (43.2) | 5,368 (42.9) | 17,756 (42.5) | 24,840 (44.0) | 81,696 (43.3) | <0.001 |
|  | Male | 44,392 (56.8) | 7,140 (57.1) | 23,980 (57.5) | 31,628 (56.0) | 107,140 (56.7) |  |
| Specimen | Upper resp. | 76,254 (97.6) | 12,281 (98.2) | 41,390 (99.2) | 56,362 (99.8) | 186,287 (98.7) | <0.001 |
|  | Lower resp. | 1,870 (2.4) | 227 (1.8) | 346 (0.8) | 106 (0.2) | 2,549 (1.3) |  |
| Age group | 0-28 d | 778 (1.0) | 243 (1.9) | 315 (0.8) | 253 (0.4) | 1,589 (0.8) | <0.001 |
|  | 29 d-23 mo | 27,621 (35.4) | 5,399 (43.2) | 9,469 (22.7) | 9,831 (17.4) | 52,320 (27.7) |  |
|  | 2-4 y | 27,114 (34.7) | 4,181 (33.4) | 15,514 (37.2) | 17,455 (30.9) | 64,264 (34.0) |  |
|  | 5-9 y | 18,127 (23.2) | 1,970 (15.7) | 12,576 (30.1) | 20,870 (37.0) | 53,543 (28.4) |  |
|  | 10-17 y | 4,484 (5.7) | 715 (5.7) | 3,862 (9.3) | 8,059 (14.3) | 17,120 (9.1) |  |
| Year | 2018 | 25,490 (32.6) | 0 (0.0) | 0 (0.0) | 0 (0.0) | 25,490 (13.5) | <0.001 |
|  | 2019 | 47,967 (61.4) | 0 (0.0) | 0 (0.0) | 0 (0.0) | 47,967 (25.4) |  |
|  | 2020 | 4,667 (6.0) | 11,979 (95.8) | 0 (0.0) | 0 (0.0) | 16,646 (8.8) |  |
|  | 2021 | 0 (0.0) | 529 (4.2) | 9,446 (22.6) | 0 (0.0) | 9,975 (5.3) |  |
|  | 2022 | 0 (0.0) | 0 (0.0) | 32,290 (77.4) | 206 (0.4) | 32,496 (17.2) |  |
|  | 2023 | 0 (0.0) | 0 (0.0) | 0 (0.0) | 56,262 (99.6) | 56,262 (29.8) |  |
| Length of stay, d (inpatients) | Median [IQR] | 8 [6–11] | 7 [6–10] | 7 [6–10] | 8 [6–11] | 8 [6–11] | <0.001 |
|  | Inpatients with data, n | 4,726 | 594 | 991 | 846 | 7,157 |  |
| Hospital cost, ¥ (inpatients) | Median [IQR] | 7500 [4901–14287] | 6708 [4226–12538] | 6509 [4087–11905] | 8477 [5398–15118] | 7419 [4744–13859] | <0.001 |
|  | Inpatients with data, n | 4,726 | 594 | 991 | 846 | 7,157 |  |

*Values are n (%) unless stated. P from chi-square (categorical) or Kruskal–Wallis (continuous). Analysis unit: patient-period (one record per patient per period); unique children also shown. Length of stay and hospital cost are restricted to inpatients with observed data (outpatients have no admission).*

**Table S4. Co-infection patterns by pandemic period (among co-infected patient-periods).**

| **Co-infection pattern** | **Pre-pandemic** | **Pandemic-I** | **Pandemic-II** | **Post-pandemic** | **Overall** | ***P*** |
| --- | --- | --- | --- | --- | --- | --- |
| Co-infected, n | 1,281 | 100 | 649 | 999 | 3,029 |  |
| Bacterial–Bacterial | 193 (15.1) | 49 (49.0) | 166 (25.6) | 56 (5.6) | 464 (15.3) | <0.001 |
| Bacterial–Viral | 219 (17.1) | 27 (27.0) | 270 (41.6) | 193 (19.3) | 709 (23.4) | <0.001 |
| Bacterial–Influenza | 78 (6.1) | 0 (0.0) | 4 (0.6) | 229 (22.9) | 311 (10.3) | <0.001 |
| Viral–Viral | 148 (11.6) | 14 (14.0) | 93 (14.3) | 62 (6.2) | 317 (10.5) | <0.001 |
| Viral–Influenza | 22 (1.7) | 0 (0.0) | 9 (1.4) | 9 (0.9) | 40 (1.3) | 0.235 |
| Influenza A–B | 785 (61.3) | 21 (21.0) | 194 (29.9) | 505 (50.6) | 1505 (49.7) | <0.001 |

*Co-infection = patient-period positive for >=2 pathogens. Bacterial group includes atypical bacteria (Mp, TB). Percentages are of co-infected in that column and may overlap (non-mutually-exclusive). P from chi-square. Influenza A-B (dual antigen positivity) is ~half of all co-infections and may partly reflect rapid-antigen cross-reactivity rather than true dual infection.*

**Table S5. Co-infection patterns by age group (among co-infected patient-periods).**

| **Co-infection pattern** | **0-28 d** | **29 d-23 mo** | **2-4 y** | **5-9 y** | **10-17 y** | **Overall** | ***P*** |
| --- | --- | --- | --- | --- | --- | --- | --- |
| Co-infected, n | 83 | 1,187 | 626 | 919 | 214 | 3,029 |  |
| Bacterial–Bacterial | 35 (42.2) | 369 (31.1) | 31 (5.0) | 23 (2.5) | 6 (2.8) | 464 (15.3) | <0.001 |
| Bacterial–Viral | 33 (39.8) | 474 (39.9) | 96 (15.3) | 83 (9.0) | 23 (10.7) | 709 (23.4) | <0.001 |
| Bacterial–Influenza | 12 (14.5) | 70 (5.9) | 38 (6.1) | 161 (17.5) | 30 (14.0) | 311 (10.3) | <0.001 |
| Viral–Viral | 0 (0.0) | 208 (17.5) | 83 (13.3) | 22 (2.4) | 4 (1.9) | 317 (10.5) | <0.001 |
| Viral–Influenza | 1 (1.2) | 17 (1.4) | 17 (2.7) | 4 (0.4) | 1 (0.5) | 40 (1.3) | 0.003 |
| Influenza A–B | 6 (7.2) | 266 (22.4) | 423 (67.6) | 651 (70.8) | 159 (74.3) | 1505 (49.7) | <0.001 |

*Co-infection = patient-period positive for >=2 pathogens. Bacterial group includes atypical bacteria (Mp, TB). Percentages are of co-infected in that column and may overlap (non-mutually-exclusive). P from chi-square. Influenza A-B (dual antigen positivity) is ~half of all co-infections and may partly reflect rapid-antigen cross-reactivity rather than true dual infection.*

**Table S6. Crude odds ratio of positivity, each pandemic period vs pre-pandemic, by pathogen.**

| **Group** | **Pathogen** | **Pandemic-I OR (95% CI)** | ***P*** | **Pandemic-II OR (95% CI)** | ***P*** | **Post OR (95% CI)** | ***P*** |
| --- | --- | --- | --- | --- | --- | --- | --- |
| Bacteria | *A. baumannii* | 1.84 (1.11–3.03) | 0.017 | 1.28 (0.87–1.89) | 0.209 | 0.93 (0.57–1.49) | 0.753 |
|  | *M. catarrhalis* | 1.52 (1.18–1.97) | 0.001 | 1.19 (0.98–1.44) | 0.075 | 0.79 (0.63–1.01) | 0.060 |
|  | *E. coli* | 1.37 (1.06–1.75) | 0.014 | 0.97 (0.80–1.17) | 0.755 | 0.85 (0.68–1.06) | 0.142 |
|  | *H. influenzae* | 0.58 (0.43–0.80) | <0.001 | 1.50 (1.29–1.74) | <0.001 | 0.70 (0.57–0.87) | <0.001 |
|  | *K. pneumoniae* | 1.20 (0.84–1.70) | 0.317 | 0.78 (0.60–1.02) | 0.070 | 0.58 (0.41–0.82) | 0.002 |
|  | *P. aeruginosa* | 0.93 (0.68–1.27) | 0.645 | 0.63 (0.50–0.79) | <0.001 | 0.38 (0.28–0.53) | <0.001 |
|  | *S. aureus* | 1.58 (1.30–1.93) | <0.001 | 1.53 (1.33–1.76) | <0.001 | 1.36 (1.16–1.60) | <0.001 |
|  | *S. pneumoniae* | 1.06 (0.83–1.35) | 0.650 | 1.27 (1.09–1.48) | 0.003 | 0.78 (0.64–0.96) | 0.016 |
|  | Other bacteria | 0.88 (0.72–1.07) | 0.204 | 0.58 (0.51–0.68) | <0.001 | 0.39 (0.32–0.47) | <0.001 |
| Atypical | *M. pneumoniae* | 0.16 (0.06–0.44) | <0.001 | 0.56 (0.39–0.80) | 0.002 | 2.24 (1.81–2.77) | <0.001 |
|  | *M. tuberculosis* | — |  | — |  | — |  |
| Virus | Adenovirus | — |  | 0.25 (0.18–0.35) | <0.001 | 0.13 (0.09–0.19) | <0.001 |
|  | Bocavirus | 0.68 (0.23–1.98) | 0.474 | 0.86 (0.50–1.50) | 0.601 | 0.96 (0.52–1.75) | 0.887 |
|  | CMV | 1.18 (0.75–1.86) | 0.473 | 0.58 (0.45–0.76) | <0.001 | 0.44 (0.32–0.61) | <0.001 |
|  | EBV | 0.44 (0.20–0.99) | 0.046 | 1.12 (0.82–1.54) | 0.475 | 1.67 (1.21–2.30) | 0.002 |
|  | Enterovirus | 0.53 (0.25–1.12) | 0.098 | 0.49 (0.33–0.73) | <0.001 | 0.36 (0.21–0.61) | <0.001 |
|  | Rhinovirus | 1.01 (0.54–1.88) | 0.976 | 1.27 (0.88–1.83) | 0.206 | 0.49 (0.29–0.83) | 0.008 |
|  | RSV | 0.50 (0.30–0.85) | 0.010 | 0.35 (0.29–0.43) | <0.001 | 0.53 (0.43–0.64) | <0.001 |
| Influenza | Influenza A | 0.22 (0.20–0.24) | <0.001 | 1.40 (1.37–1.45) | <0.001 | 1.78 (1.74–1.83) | <0.001 |
|  | Influenza B | 0.07 (0.06–0.09) | <0.001 | 0.95 (0.91–1.00) | 0.059 | 1.22 (1.17–1.27) | <0.001 |

*Reference: pre-pandemic. '—' = not estimable (sparse). Crude (unadjusted) OR from logistic regression.*

**Table S7. Univariable and multivariable logistic regression for RSV positivity.**

| **Variable** | **Level** | **N** | **Positive n (%)** | **cOR (95% CI)** | ***P (crude)*** | **aOR (95% CI)** | ***P (adj)*** |
| --- | --- | --- | --- | --- | --- | --- | --- |
| Pandemic period | Pre-pandemic | 682 | 149 (21.8) | 1.00 (Ref) |  | 1.00 (Ref) |  |
|  | Pandemic-I | 146 | 18 (12.3) | 0.50 (0.30–0.85) | 0.010 | 0.54 (0.31–0.92) | 0.027 |
|  | Pandemic-II | 7,240 | 643 (8.9) | 0.35 (0.29–0.43) | <0.001 | 1.07 (0.87–1.32) | 0.522 |
|  | Post-pandemic | 5,790 | 743 (12.8) | 0.53 (0.43–0.64) | <0.001 | 1.31 (1.06–1.62) | 0.014 |
| Age group | 0-28 d | 8,222 | 135 (1.6) | 0.04 (0.03–0.04) | <0.001 | 0.04 (0.03–0.05) | <0.001 |
|  | 29 d-23 mo | 3,298 | 1026 (31.1) | 1.00 (Ref) |  | 1.00 (Ref) |  |
|  | 2-4 y | 1,541 | 365 (23.7) | 0.69 (0.60–0.79) | <0.001 | 0.66 (0.57–0.76) | <0.001 |
|  | 5-9 y | 669 | 25 (3.7) | 0.09 (0.06–0.13) | <0.001 | 0.08 (0.05–0.12) | <0.001 |
|  | 10-17 y | 128 | 2 (1.6) | 0.04 (0.01–0.14) | <0.001 | 0.03 (0.01–0.12) | <0.001 |
| Sex | Female | 5,742 | 632 (11.0) | 1.00 (Ref) |  | 1.00 (Ref) |  |
|  | Male | 8,116 | 921 (11.3) | 1.03 (0.93–1.15) | 0.530 | 0.85 (0.76–0.96) | 0.010 |
| Care setting | Outpatient | 474 | 177 (37.3) | 1.00 (Ref) |  | 1.00 (Ref) |  |
|  | Inpatient | 13,384 | 1376 (10.3) | 0.19 (0.16–0.23) | <0.001 | 0.52 (0.42–0.65) | <0.001 |
| Hospital campus | Panyu (PY) | 10,305 | 729 (7.1) | 1.00 (Ref) |  | 1.00 (Ref) |  |
|  | YX | 3,553 | 824 (23.2) | 3.97 (3.56–4.42) | <0.001 | 1.10 (0.96–1.26) | 0.219 |

*cOR, crude OR; aOR, adjusted OR (all variables simultaneously); CI, confidence interval. Reference: pre-pandemic; 29 d-23 mo; female; outpatient; Panyu. Specimen type excluded from the model (mediator).*

**Table S8. Univariable and multivariable logistic regression for Influenza A positivity.**

| **Variable** | **Level** | **N** | **Positive n (%)** | **cOR (95% CI)** | ***P (crude)*** | **aOR (95% CI)** | ***P (adj)*** |
| --- | --- | --- | --- | --- | --- | --- | --- |
| Pandemic period | Pre-pandemic | 78,115 | 14970 (19.2) | 1.00 (Ref) |  | 1.00 (Ref) |  |
|  | Pandemic-I | 12,508 | 623 (5.0) | 0.22 (0.20–0.24) | <0.001 | 0.23 (0.21–0.25) | <0.001 |
|  | Pandemic-II | 41,735 | 10426 (25.0) | 1.40 (1.37–1.45) | <0.001 | 1.26 (1.23–1.30) | <0.001 |
|  | Post-pandemic | 56,467 | 16770 (29.7) | 1.78 (1.74–1.83) | <0.001 | 1.52 (1.48–1.56) | <0.001 |
| Age group | 0-28 d | 1,589 | 36 (2.3) | 0.14 (0.10–0.20) | <0.001 | 0.35 (0.25–0.49) | <0.001 |
|  | 29 d-23 mo | 52,319 | 7275 (13.9) | 1.00 (Ref) |  | 1.00 (Ref) |  |
| p | 2-4 y | 64,259 | 14796 (23.0) | 1.85 (1.80–1.91) | <0.001 | 1.66 (1.61–1.71) | <0.001 |
|  | 5-9 y | 53,538 | 16075 (30.0) | 2.66 (2.58–2.74) | <0.001 | 2.19 (2.12–2.26) | <0.001 |
|  | 10-17 y | 17,120 | 4607 (26.9) | 2.28 (2.19–2.38) | <0.001 | 1.81 (1.74–1.89) | <0.001 |
| Sex | Female | 81,692 | 18130 (22.2) | 1.00 (Ref) |  | 1.00 (Ref) |  |
|  | Male | 107,133 | 24659 (23.0) | 1.05 (1.03–1.07) | <0.001 | 1.07 (1.05–1.10) | <0.001 |
| Care setting | Outpatient | 181,668 | 42461 (23.4) | 1.00 (Ref) |  | 1.00 (Ref) |  |
|  | Inpatient | 7,157 | 328 (4.6) | 0.16 (0.14–0.18) | <0.001 | 0.25 (0.22–0.28) | <0.001 |
| Hospital campus | Panyu (PY) | 120,528 | 26721 (22.2) | 1.00 (Ref) |  | 1.00 (Ref) |  |
|  | YX | 68,297 | 16068 (23.5) | 1.08 (1.06–1.10) | <0.001 | 1.10 (1.07–1.12) | <0.001 |

*cOR, crude OR; aOR, adjusted OR (all variables simultaneously); CI, confidence interval. Reference: pre-pandemic; 29 d-23 mo; female; outpatient; Panyu. Specimen type excluded from the model (mediator).*

**Table S9. Univariable and multivariable logistic regression for Influenza B positivity.**

| **Variable** | **Level** | **N** | **Positive n (%)** | **cOR (95% CI)** | ***P (crude)*** | **aOR (95% CI)** | ***P (adj)*** |
| --- | --- | --- | --- | --- | --- | --- | --- |
| Pandemic period | Pre-pandemic | 78,123 | 5088 (6.5) | 1.00 (Ref) |  | 1.00 (Ref) |  |
|  | Pandemic-I | 12,508 | 63 (0.5) | 0.07 (0.06–0.09) | <0.001 | 0.08 (0.06–0.10) | <0.001 |
|  | Pandemic-II | 41,736 | 2601 (6.2) | 0.95 (0.91–1.00) | 0.059 | 0.84 (0.80–0.88) | <0.001 |
|  | Post-pandemic | 56,466 | 4432 (7.8) | 1.22 (1.17–1.27) | <0.001 | 0.95 (0.91–1.00) | 0.025 |
| Age group | 0-28 d | 1,588 | 68 (4.3) | 1.05 (0.82–1.34) | 0.707 | 1.61 (1.23–2.09) | <0.001 |
|  | 29 d-23 mo | 52,320 | 2141 (4.1) | 1.00 (Ref) |  | 1.00 (Ref) |  |
|  | 2-4 y | 64,263 | 2639 (4.1) | 1.00 (0.95–1.06) | 0.902 | 0.96 (0.91–1.02) | 0.225 |
|  | 5-9 y | 53,543 | 5295 (9.9) | 2.57 (2.44–2.71) | <0.001 | 2.42 (2.29–2.55) | <0.001 |
|  | 10-17 y | 17,119 | 2041 (11.9) | 3.17 (2.98–3.38) | <0.001 | 3.02 (2.83–3.23) | <0.001 |
| Sex | Female | 81,693 | 5057 (6.2) | 1.00 (Ref) |  | 1.00 (Ref) |  |
|  | Male | 107,140 | 7127 (6.7) | 1.08 (1.04–1.12) | <0.001 | 1.10 (1.06–1.14) | <0.001 |
| Care setting | Outpatient | 181,677 | 11954 (6.6) | 1.00 (Ref) |  | 1.00 (Ref) |  |
|  | Inpatient | 7,156 | 230 (3.2) | 0.47 (0.41–0.54) | <0.001 | 0.60 (0.52–0.70) | <0.001 |
| Hospital campus | Panyu (PY) | 120,531 | 8494 (7.0) | 1.00 (Ref) |  | 1.00 (Ref) |  |
|  | YX | 68,302 | 3690 (5.4) | 0.75 (0.72–0.78) | <0.001 | 0.74 (0.71–0.77) | <0.001 |

*cOR, crude OR; aOR, adjusted OR (all variables simultaneously); CI, confidence interval. Reference: pre-pandemic; 29 d-23 mo; female; outpatient; Panyu. Specimen type excluded from the model (mediator).*

**Table S10. Univariable and multivariable logistic regression for M. pneumoniae positivity.**

| **Variable** | **Level** | **N** | **Positive n (%)** | **cOR (95% CI)** | ***P (crude)*** | **aOR (95% CI)** | ***P (adj)*** |
| --- | --- | --- | --- | --- | --- | --- | --- |
| Pandemic period | Pre-pandemic | 828 | 103 (12.4) | 1.00 (Ref) |  | 1.00 (Ref) |  |
|  | Pandemic-I | 182 | 4 (2.2) | 0.16 (0.06–0.44) | <0.001 | 0.20 (0.07–0.55) | 0.002 |
|  | Pandemic-II | 652 | 48 (7.4) | 0.56 (0.39–0.80) | 0.002 | 0.54 (0.37–0.78) | 0.001 |
|  | Post-pandemic | 7,739 | 1868 (24.1) | 2.24 (1.81–2.77) | <0.001 | 1.99 (1.53–2.59) | <0.001 |
| Age group | 0-28 d | 300 | 11 (3.7) | 0.43 (0.23–0.80) | 0.008 | 0.53 (0.28–1.00) | 0.049 |
|  | 29 d-23 mo | 1,753 | 143 (8.2) | 1.00 (Ref) |  | 1.00 (Ref) |  |
|  | 2-4 y | 2,731 | 414 (15.2) | 2.01 (1.65–2.46) | <0.001 | 2.18 (1.75–2.71) | <0.001 |
|  | 5-9 y | 3,530 | 1222 (34.6) | 5.96 (4.96–7.17) | <0.001 | 6.38 (5.18–7.86) | <0.001 |
|  | 10-17 y | 1,087 | 233 (21.4) | 3.07 (2.45–3.84) | <0.001 | 3.29 (2.57–4.20) | <0.001 |
| Sex | Female | 4,101 | 909 (22.2) | 1.00 (Ref) |  | 1.00 (Ref) |  |
|  | Male | 5,300 | 1114 (21.0) | 0.93 (0.85–1.03) | 0.180 | 1.01 (0.91–1.12) | 0.839 |
| Care setting | Outpatient | 7,212 | 1672 (23.2) | 1.00 (Ref) |  | 1.00 (Ref) |  |
|  | Inpatient | 2,189 | 351 (16.0) | 0.63 (0.56–0.72) | <0.001 | 2.38 (1.98–2.86) | <0.001 |
| Hospital campus | Panyu (PY) | 3,386 | 702 (20.7) | 1.00 (Ref) |  | 1.00 (Ref) |  |
|  | YX | 6,015 | 1321 (22.0) | 1.08 (0.97–1.19) | 0.164 | 1.00 (0.89–1.11) | 0.945 |

*cOR, crude OR; aOR, adjusted OR (all variables simultaneously); CI, confidence interval. Reference: pre-pandemic; 29 d-23 mo; female; outpatient; Panyu. Specimen type excluded from the model (mediator).*

**Table S11. Sensitivity - adjusted OR restricted to inpatients (more consistent testing indications).**

| **Pathogen** | **Period** | **pos/tested** | **aOR (95% CI)** | ***P*** |
| --- | --- | --- | --- | --- |
| RSV | Pre-pandemic | 149/674 | 1.00 (Ref) |  |
|  | Pandemic-I | 18/146 | 0.53 (0.30–0.91) | 0.022 |
|  | Pandemic-II | 602/7103 | 1.09 (0.88–1.35) | 0.428 |
|  | Post-pandemic | 607/5461 | 1.29 (1.03–1.60) | 0.025 |
| Influenza A | Pre-pandemic | 236/4726 | 1.00 (Ref) |  |
|  | Pandemic-I | 6/594 | 0.23 (0.10–0.52) | <0.001 |
|  | Pandemic-II | 31/991 | 0.64 (0.44–0.95) | 0.025 |
|  | Post-pandemic | 55/846 | 1.37 (1.01–1.87) | 0.042 |
| Influenza B | Pre-pandemic | 202/4725 | 1.00 (Ref) |  |
|  | Pandemic-I | 2/594 | NE |  |
|  | Pandemic-II | 20/991 | 0.43 (0.26–0.69) | <0.001 |
|  | Post-pandemic | 6/846 | 0.15 (0.06–0.34) | <0.001 |
| M. pneumoniae | Pre-pandemic | 90/702 | 1.00 (Ref) |  |
|  | Pandemic-I | 4/177 | NE |  |
|  | Pandemic-II | 47/634 | 0.46 (0.30–0.70) | <0.001 |
|  | Post-pandemic | 210/676 | 1.49 (1.08–2.05) | 0.014 |
| A. baumannii | Pre-pandemic | 50/13355 | 1.00 (Ref) |  |
|  | Pandemic-I | 22/3171 | 1.87 (1.13–3.09) | 0.015 |
|  | Pandemic-II | 49/10437 | 1.52 (1.02–2.26) | 0.040 |
|  | Post-pandemic | 25/7183 | 1.31 (0.81–2.14) | 0.275 |
| M. catarrhalis | Pre-pandemic | 184/13355 | 1.00 (Ref) |  |
|  | Pandemic-I | 81/3171 | 1.94 (1.48–2.54) | <0.001 |
|  | Pandemic-II | 199/10437 | 1.57 (1.27–1.93) | <0.001 |
|  | Post-pandemic | 94/7183 | 1.26 (0.97–1.63) | 0.080 |
| E. coli | Pre-pandemic | 240/13355 | 1.00 (Ref) |  |
|  | Pandemic-I | 80/3171 | 1.40 (1.08–1.81) | 0.011 |
|  | Pandemic-II | 189/10437 | 1.43 (1.18–1.73) | <0.001 |
|  | Post-pandemic | 113/7183 | 1.46 (1.16–1.83) | 0.001 |
| H. influenzae | Pre-pandemic | 263/13355 | 1.00 (Ref) |  |
|  | Pandemic-I | 43/3171 | 0.70 (0.50–0.97) | 0.031 |
|  | Pandemic-II | 355/10437 | 2.25 (1.91–2.64) | <0.001 |
|  | Post-pandemic | 116/7183 | 1.23 (0.98–1.53) | 0.071 |
| P. aeruginosa | Pre-pandemic | 218/13355 | 1.00 (Ref) |  |
|  | Pandemic-I | 46/3171 | 0.89 (0.64–1.23) | 0.471 |
|  | Pandemic-II | 105/10437 | 0.81 (0.64–1.03) | 0.081 |
|  | Post-pandemic | 46/7183 | 0.61 (0.44–0.83) | 0.002 |
| S. aureus | Pre-pandemic | 354/13355 | 1.00 (Ref) |  |
|  | Pandemic-I | 133/3171 | 1.68 (1.37–2.06) | <0.001 |
|  | Pandemic-II | 422/10437 | 1.62 (1.40–1.87) | <0.001 |
|  | Post-pandemic | 261/7183 | 1.39 (1.17–1.64) | <0.001 |
| S. pneumoniae | Pre-pandemic | 300/13355 | 1.00 (Ref) |  |
|  | Pandemic-I | 80/3171 | 1.18 (0.91–1.51) | 0.207 |
|  | Pandemic-II | 313/10437 | 1.16 (0.99–1.36) | 0.073 |
|  | Post-pandemic | 136/7183 | 0.78 (0.63–0.96) | 0.020 |
| Other bacteria | Pre-pandemic | 589/13355 | 1.00 (Ref) |  |
|  | Pandemic-I | 121/3171 | 0.88 (0.72–1.07) | 0.209 |
|  | Pandemic-II | 272/10437 | 0.65 (0.56–0.75) | <0.001 |
|  | Post-pandemic | 126/7183 | 0.47 (0.38–0.57) | <0.001 |

*Inpatients only (43,689 patient-periods), where testing is more clinically driven and stable across periods. Adjusted for age, sex and hospital campus with cluster-robust SE by child. NE = not estimable (sparse). Reference: pre-pandemic.*

**Table S12. Sensitivity - bacterial adjusted OR with vs without specimen type in the model.**

| **Pathogen** | **Period** | **Primary aOR (95% CI)** | ***P*** | **With specimen type aOR (95% CI)** | ***P*** |
| --- | --- | --- | --- | --- | --- |
| A. baumannii | Pandemic-I | 1.85 (1.12–3.05) | 0.017 | 1.69 (1.02–2.78) | 0.041 |
|  | Pandemic-II | 1.55 (1.04–2.30) | 0.031 | 1.49 (1.01–2.21) | 0.046 |
|  | Post-pandemic | 1.31 (0.81–2.14) | 0.275 | 2.31 (1.35–3.98) | 0.002 |
| M. catarrhalis | Pandemic-I | 1.73 (1.33–2.24) | <0.001 | 1.62 (1.25–2.11) | <0.001 |
|  | Pandemic-II | 1.53 (1.25–1.86) | <0.001 | 1.50 (1.23–1.83) | <0.001 |
|  | Post-pandemic | 1.21 (0.94–1.56) | 0.131 | 1.77 (1.37–2.29) | <0.001 |
| E. coli | Pandemic-I | 1.40 (1.09–1.80) | 0.009 | 1.31 (1.02–1.69) | 0.034 |
|  | Pandemic-II | 1.43 (1.18–1.73) | <0.001 | 1.43 (1.18–1.73) | <0.001 |
|  | Post-pandemic | 1.48 (1.18–1.84) | <0.001 | 4.71 (3.75–5.92) | <0.001 |
| H. influenzae | Pandemic-I | 0.66 (0.48–0.90) | 0.009 | 0.61 (0.44–0.84) | 0.003 |
|  | Pandemic-II | 2.21 (1.89–2.58) | <0.001 | 2.20 (1.87–2.57) | <0.001 |
|  | Post-pandemic | 1.20 (0.97–1.49) | 0.093 | 2.26 (1.81–2.82) | <0.001 |
| K. pneumoniae | Pandemic-I | 1.25 (0.88–1.78) | 0.221 | 1.14 (0.80–1.63) | 0.469 |
|  | Pandemic-II | 0.98 (0.75–1.28) | 0.875 | 0.95 (0.72–1.24) | 0.694 |
|  | Post-pandemic | 0.85 (0.60–1.20) | 0.353 | 1.55 (1.06–2.28) | 0.025 |
| P. aeruginosa | Pandemic-I | 0.94 (0.69–1.29) | 0.696 | 0.85 (0.62–1.17) | 0.312 |
|  | Pandemic-II | 0.84 (0.66–1.06) | 0.140 | 0.81 (0.64–1.02) | 0.075 |
|  | Post-pandemic | 0.61 (0.44–0.83) | 0.002 | 1.84 (1.30–2.62) | <0.001 |
| S. aureus | Pandemic-I | 1.65 (1.35–2.02) | <0.001 | 1.59 (1.30–1.94) | <0.001 |
|  | Pandemic-II | 1.60 (1.39–1.84) | <0.001 | 1.63 (1.41–1.87) | <0.001 |
|  | Post-pandemic | 1.37 (1.16–1.62) | <0.001 | 1.79 (1.50–2.12) | <0.001 |
| S. pneumoniae | Pandemic-I | 1.14 (0.89–1.46) | 0.294 | 1.10 (0.86–1.41) | 0.463 |
|  | Pandemic-II | 1.15 (0.98–1.35) | 0.079 | 1.14 (0.98–1.34) | 0.098 |
|  | Post-pandemic | 0.77 (0.62–0.94) | 0.012 | 0.87 (0.70–1.08) | 0.198 |
| Other bacteria | Pandemic-I | 0.90 (0.74–1.10) | 0.319 | 0.85 (0.69–1.03) | 0.099 |
|  | Pandemic-II | 0.65 (0.56–0.75) | <0.001 | 0.64 (0.55–0.74) | <0.001 |
|  | Post-pandemic | 0.47 (0.38–0.57) | <0.001 | 0.66 (0.53–0.82) | <0.001 |

*Primary model excludes specimen type (a near-deterministic mediator of culture yield that shifted across periods); adding it markedly inflates/reverses several estimates (e.g. E. coli), illustrating over-adjustment. All models use cluster-robust SE by child. Reference: pre-pandemic.*

**Table S13. Sensitivity - co-infection by period EXCLUDING influenza A–B dual-antigen positivity.**

| **Co-infection pattern** | **Pre-pandemic** | **Pandemic-I** | **Pandemic-II** | **Post-pandemic** | **Overall** | ***P*** |
| --- | --- | --- | --- | --- | --- | --- |
| Co-infected, n | 504 | 79 | 456 | 498 | 1,537 |  |
| Bacterial–Bacterial | 193 (38.3) | 49 (62.0) | 166 (36.4) | 56 (11.2) | 464 (30.2) | <0.001 |
| Bacterial–Viral | 219 (43.5) | 27 (34.2) | 270 (59.2) | 193 (38.8) | 709 (46.1) | <0.001 |
| Bacterial–Influenza | 78 (15.5) | 0 (0.0) | 4 (0.9) | 229 (46.0) | 311 (20.2) | <0.001 |
| Viral–Viral | 148 (29.4) | 14 (17.7) | 93 (20.4) | 62 (12.4) | 317 (20.6) | <0.001 |
| Viral–Influenza | 22 (4.4) | 0 (0.0) | 9 (2.0) | 9 (1.8) | 40 (2.6) | 0.017 |

*Influenza counted as one positive regardless of A/B, and the influenza A–B category removed (possible antigen cross-reactivity). Co-infected patient-periods fall from 3,029 to 1,537. Percentages are of co-infected per column and may overlap. P from chi-square.*

**Table S14. Number of patients tested per pathogen and period (testing-volume change).**

| **Group** | **Pathogen** | **Pre-pandemic** | **Pandemic-I** | **Pandemic-II** | **Post-pandemic** | **Post : Pre** |
| --- | --- | --- | --- | --- | --- | --- |
| Bacteria | A. baumannii | 14,051 | 3,245 | 10,537 | 7,292 | 0.5× |
|  | M. catarrhalis | 14,051 | 3,245 | 10,537 | 7,292 | 0.5× |
|  | E. coli | 14,051 | 3,245 | 10,537 | 7,292 | 0.5× |
|  | H. influenzae | 14,051 | 3,245 | 10,537 | 7,292 | 0.5× |
|  | K. pneumoniae | 14,051 | 3,245 | 10,537 | 7,292 | 0.5× |
|  | P. aeruginosa | 14,051 | 3,245 | 10,537 | 7,292 | 0.5× |
|  | S. aureus | 14,051 | 3,245 | 10,537 | 7,292 | 0.5× |
|  | S. pneumoniae | 14,051 | 3,245 | 10,537 | 7,292 | 0.5× |
|  | Other bacteria | 14,051 | 3,245 | 10,537 | 7,292 | 0.5× |
| Atypical | M. pneumoniae | 828 | 182 | 652 | 7,739 | 9.3× |
|  | M. tuberculosis | 698 | 133 | 534 | 338 | 0.5× |
| Virus | Adenovirus | 489 | 178 | 1,356 | 2,097 | 4.3× |
|  | Bocavirus | 454 | 94 | 484 | 321 | 0.7× |
|  | CMV | 642 | 92 | 490 | 322 | 0.5× |
|  | EBV | 638 | 90 | 489 | 369 | 0.6× |
|  | Enterovirus | 787 | 119 | 576 | 348 | 0.4× |
|  | Rhinovirus | 468 | 114 | 508 | 328 | 0.7× |
|  | RSV | 682 | 146 | 7,240 | 5,790 | 8.5× |
| Influenza | Influenza A | 78,115 | 12,508 | 41,735 | 56,467 | 0.7× |
|  | Influenza B | 78,123 | 12,508 | 41,736 | 56,466 | 0.7× |

*Denominators (patients tested) shifted markedly across periods as testing moved from selective to routine — notably RSV (8.5×) and M. pneumoniae (9.3×). This motivates the comparable-denominator analysis (Table S15, Fig S4) and interpreting positivity as test positivity among tested children, not incidence.*

**Table S15. Comparable-denominator sensitivity-adjusted OR (each period vs pre-pandemic) under progressively stable testing indications.**

| **Pathogen** | **Population** | **Pre-pandemic pos/n** | **Pandemic-I pos/n; aOR (95% CI)** | **Pandemic-II pos/n; aOR (95% CI)** | **Post-pandemic pos/n; aOR (95% CI)** |
| --- | --- | --- | --- | --- | --- |
| RSV | Full cohort | 149/682 | 18/146; 0.54 (0.31–0.93)* | 643/7240; 1.07 (0.87–1.33) | 743/5790; 1.31 (1.06–1.63)* |
|  | Inpatients | 149/674 | 18/146; 0.53 (0.30–0.91)* | 602/7103; 1.09 (0.88–1.35) | 607/5461; 1.29 (1.03–1.60)* |
|  | Inpatients + lower-resp. | 148/671 | 18/137; 0.53 (0.31–0.91)* | 356/1447; 1.33 (1.05–1.68)* | 105/375; 2.20 (1.61–3.00)*** |
| Influenza A | Full cohort | 14970/78115 | 623/12508; 0.23 (0.21–0.25)*** | 10426/41735; 1.26 (1.23–1.30)*** | 16770/56467; 1.52 (1.48–1.56)*** |
|  | Inpatients | 236/4726 | 6/594; 0.23 (0.10–0.52)*** | 31/991; 0.64 (0.44–0.95)* | 55/846; 1.37 (1.01–1.87)* |
|  | Inpatients + lower-resp. | 76/1837 | 0/225; NE | 6/331; 0.45 (0.19–1.08) | 9/106; 1.81 (0.89–3.69) |
| Influenza B | Full cohort | 5088/78123 | 63/12508; 0.08 (0.06–0.10)*** | 2601/41736; 0.84 (0.80–0.88)*** | 4432/56466; 0.95 (0.91–0.99)* |
|  | Inpatients | 202/4725 | 2/594; NE | 20/991; 0.43 (0.26–0.69)*** | 6/846; 0.15 (0.06–0.34)*** |
|  | Inpatients + lower-resp. | 123/1836 | 0/225; NE | 8/331; 0.33 (0.16–0.68)** | 1/106; NE |
| M. pneumoniae | Full cohort | 103/828 | 4/182; NE | 48/652; 0.54 (0.37–0.78)** | 1868/7739; 1.99 (1.54–2.57)*** |
|  | Inpatients | 90/702 | 4/177; NE | 47/634; 0.46 (0.30–0.70)*** | 210/676; 1.49 (1.08–2.05)* |
|  | Inpatients + lower-resp. | 89/693 | 4/171; NE | 47/605; 0.46 (0.30–0.70)*** | 110/366; 1.76 (1.23–2.51)** |

*Adjusted for age, sex and hospital campus (cluster-robust by child). The post-pandemic rebound for RSV, influenza A and M. pneumoniae persists — and for RSV/Mp strengthens — as the denominator is restricted to clinically indicated testing, indicating it is not an artifact of denominator expansion. NE = not estimable (sparse; e.g. all-upper specimens for influenza). *P<0.05, **P<0.01, ***P<0.001.*

**Table S16. Specimen-specific bacterial positivity (culture yield by specimen type).**

| **Pathogen** | **Upper resp. pos/n (%)** | **Lower resp. pos/n (%)** | **Lower : Upper yield** |
| --- | --- | --- | --- |
| *A. baumannii* | 40/21,743 (0.18) | 109/13,382 (0.81) | 4.4× |
| *M. catarrhalis* | 212/21,743 (0.98) | 403/13,382 (3.01) | 3.1× |
| *E. coli* | 104/21,743 (0.48) | 554/13,382 (4.14) | 8.7× |
| *H. influenzae* | 215/21,743 (0.99) | 648/13,382 (4.84) | 4.9× |
| *K. pneumoniae* | 75/21,743 (0.34) | 239/13,382 (1.79) | 5.2× |
| *P. aeruginosa* | 46/21,743 (0.21) | 385/13,382 (2.88) | 13.6× |
| *S. aureus* | 571/21,743 (2.63) | 626/13,382 (4.68) | 1.8× |
| *S. pneumoniae* | 511/21,743 (2.35) | 362/13,382 (2.71) | 1.2× |
| Other bacteria | 388/21,743 (1.78) | 756/13,382 (5.65) | 3.2× |

*Among bacterial-cultured patient-periods (upper-respiratory n=21,743; lower-respiratory n=13,382). Culture yield is markedly higher in lower-respiratory specimens; because the post-pandemic specimen mix shifted toward upper-respiratory, specimen type acts as a mediator, which is why it is excluded from the primary bacterial models (see Table S12).*

Table S17. Data dictionary for the analysis dataset

| Variable | Description | Coding / Unit | Missing |
| --- | --- | --- | --- |
| child_id | Anonymized child identifier (clustering key for cluster-robust SEs). Original hospital IDs replaced with sequential codes. | — | None |
| Period | Pandemic period | Pre-pandemic; Pandemic-I; Pandemic-II; Post-pandemic | None |
| Age | Age group | A.0-28D (0–28 d); B.29D-23M (29 d–23 mo); C.2-4Y (2–4 y); D.5-9Y (5–9 y); E.10-17Y (10–17 y) | None |
| Sex | Sex | Female; Male | None |
| Setting | Care setting | In (inpatient); Out (outpatient) | None |
| YQ | Hospital campus | PY (Panyu); YX | None |
| Sample | Specimen type | Upper (upper respiratory); Lower (lower respiratory) | None |
| Year | Calendar year of the record | 2018–2023 | None |
| LOS | Length of hospital stay | Days | Restricted to inpatients with observed data |
| cost | Hospital charges | CNY (¥) | Restricted to inpatients with observed data |
| tested_Bacteria | Had a bacterial culture test | 1 = yes; 0 = no | None |
| tested_Virus | Had a viral/atypical PCR test | 1 = yes; 0 = no | None |
| tested_Flu | Had an influenza antigen test | 1 = yes; 0 = no | None |
| test_<pathogen> | Tested for that pathogen in this patient-period | 1 = yes; 0 = no | None |
| pos_<pathogen> | Positive for that pathogen | 1 = positive; 0 = negative; denominator = test_<pathogen> == 1 | None |

*One row represents one patient-period (a child within one pandemic period); 230,333 rows from 197,630 unique children. The denominator for each pathogen is test_<pathogen> == 1. Bacterial pathogens were identified by routine culture; M. pneumoniae and M. tuberculosis by nucleic acid amplification; adenovirus, bocavirus, CMV, EBV, enterovirus, rhinovirus, and RSV by multiplex real-time fluorescence PCR; influenza A and B by rapid antigen testing. child_id is an anonymized sequential code used as the clustering key for cluster-robust standard errors. LOS and cost are restricted to inpatients with observed data. Exclusions (incomplete demographic or clinical data, duplicate records, and surveillance/non-clinical tests) were applied during data curation upstream of this file. Aba, A. baumannii; Bca, M. catarrhalis; Eco, E. coli; Hin, H. influenzae; Kpn, K. pneumoniae; Pae, P. aeruginosa; Sau, S. aureus; Spn, S. pneumoniae; Mp, M. pneumoniae; TB, M. tuberculosis; ADV, adenovirus; BoCV, bocavirus; hEnV, enterovirus; hRhV, rhinovirus; RSV, respiratory syncytial virus; FluA, influenza A; FluB, influenza B.*
